# Discovery of a Novel Coumarin/Thiazole Chalcone Hybrid as a Potent Dual Inhibitor of Tubulin and Carbonic Anhydrases IX & XII with Promising Anti-Proliferative Activity

**DOI:** 10.3390/molecules31060917

**Published:** 2026-03-10

**Authors:** Basima A. A. Saleem, Ashraf A. Qurtam, Mohamed Ahmed, Raed Fanoukh Aboqader Al-Aouadi, Ali Abdulrazzaq Abdulhussein Alrikabi, Helal F. Hetta, Stefan Bräse, Ghallab Alotaibi, Abdullah Alkhammash, Sara Mahmoud Farhan

**Affiliations:** 1 Department of Chemistry, College of Science, University of Mosul, Mosul 41001, Iraq; 2 Biology Department, College of Science, Imam Mohammad Ibn Saud Islamic University (IMSIU), Riyadh 11623, Saudi Arabia; 3 College of Medicine, Dhofar University, Salalah 122, Oman; 4 College of Medicine, Al-Ayen Iraqi University (AUIQ), An Nasiriyah 64001, Iraq; 5 College of Medicine, University of Thi-Qar, Nasiriyah 64001, Iraq; 6 Division of Microbiology, Immunology and Biotechnology, Department of Natural Products and Alternative Medicine, Faculty of Pharmacy, University of Tabuk, Tabuk 71491, Saudi Arabia; 7 Institute of Biological and Chemical Systems—Functional Molecular Systems (IBCS-FMS), Karlsruhe Institute of Technology (KIT), Kaiserstrasse 12, 76131 Karlsruhe, Germany; 8 Department of Pharmacology, College of Pharmacy, Al-Dawadmi Campus, Shaqra University, Shaqra 11961, Saudi Arabia; 9 Department of Microbiology and Immunology, Faculty of Pharmacy, Deraya University, New Minia 61768, Egypt

**Keywords:** coumarins, thiazole chalcones, carbonic anhydrase, tubulin, apoptosis

## Abstract

Multitarget-directed ligands offer a promising strategy for overcoming tumor complexity through simultaneous modulation of complementary oncogenic pathways. In this work, a novel (*E*)-6-(3-(4-methyl-2-thioxo-2,3-dihydrothiazol-5-yl)-3-oxoprop-1-en-1-yl)-2H-chromen-2-one (compound **6**) was synthesized and evaluated as a dual inhibitor of tubulin polymerization and tumor-associated carbonic anhydrases (CAs) IX and XII. Compound **6** displayed potent antiproliferative activity, particularly against MDA-MB-231 triple-negative breast cancer cells (IC_50_ = 0.37 µM), with excellent selectivity toward non-tumorigenic cells. Mechanistic studies demonstrated strong tubulin polymerization inhibition (IC_50_ = 3.40 ± 0.09 µM) and submicromolar inhibition of CA IX (IC_50_ = 0.102 ± 0.005 µM) and CA XII (IC_50_ = 0.213 ± 0.004 µM), accompanied by downregulation of CA-IX and CA-XII protein expression. Cellular investigations revealed pronounced G_2_/M phase arrest and apoptosis induction via mitochondrial signaling and caspase activation. Anti-angiogenic activity was supported by inhibition of endothelial migration and concentration-dependent suppression of VEGFR-2 (Tyr1175) phosphorylation in HUVEC cells. Human liver microsomal assays indicated measurable metabolic stability, while molecular docking and in silico ADMET predictions supported target engagement and drug-like properties. Collectively, these findings identify compound **6** as a promising multitarget anticancer lead integrating antimitotic, metabolic, and anti-angiogenic mechanisms.

## 1. Introduction

Multitarget-directed ligands (MTDLs) offer a rational strategy to overcome the complexity and redundancy of oncogenic signaling by embedding two or more pharmacophores within a single chemotype to modulate distinct cancer-relevant pathways [1,2]. This polypharmacological approach enhances synergy, limits resistance driven by pathway crosstalk, and simplifies pharmacokinetic profiles [3,4,5]. A particularly attractive design incorporates one element that disrupts tumor metabolic adaptation alongside another that impairs cell-cycle progression [6,7]. Among the most validated metabolic targets for such MTDLs are carbonic anhydrase (CA) isoforms IX and XII—transmembrane zinc metalloenzymes that catalyze the reversible hydration of CO_2_ to bicarbonate and protons, maintaining intracellular pH and driving extracellular acidification under hypoxia [8,9,10]. This acid-base imbalance supports tumor invasion, metastasis, and therapy resistance [11]. Unlike the ubiquitous cytosolic isoforms CA I and II, CA IX and XII are strongly upregulated in malignant tissues through HIF-1α regulation, making them highly selective and druggable targets in the tumor microenvironment [12].

Coumarins constitute a mechanistically distinct and well-established class of non-sulfonamide CA inhibitors (CAIs) that are particularly well suited for targeting these tumor-associated isoforms [13]. In contrast to classical sulfonamide-based CAIs that coordinate the active-site zinc ion, coumarins behave as “prodrug” inhibitors: upon entering the CA active site, the lactone ring undergoes enzymatic hydrolysis to generate a 2-hydroxycinnamic acid derivative, which then binds at the mouth of the catalytic cleft rather than deep within the zinc-coordination sphere [14]. This entrance-directed binding mode exploits the higher sequence and conformational variability at the active-site rim, providing a structural basis for isoform selectivity [15].

**Figure 1:**
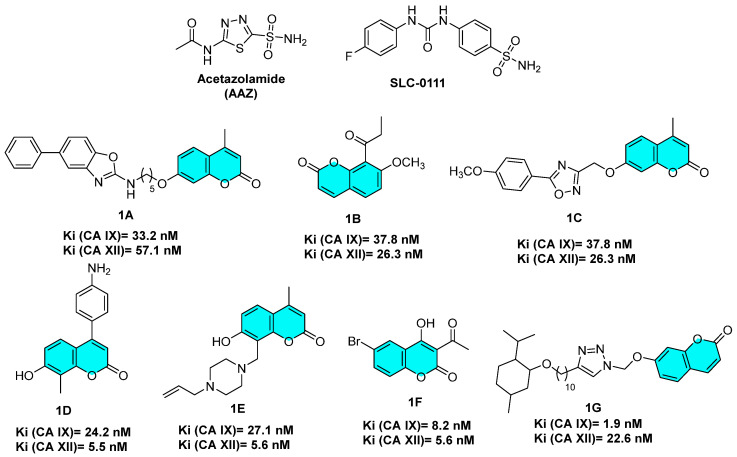
Examples of coumarin-based compounds as selective CA IX/XII inhibitors (K_i_ = inhibition constant).

Several coumarin-based compounds have shown potent and selective inhibition of CA IX and XII isoforms (Figure 1). Fuentes-Aguilar et al. identified benzoxazole–coumarin **1A** as a selective hCA IX/XII inhibitor with negligible inhibition of hCA I/II and only weak hCA IV inhibition [16], while Maresca et al. reported 7,8-disubstituted coumarin **1B** as a prototypical entrance-binding CAI that potently inhibits hCA IX/XII and essentially spares the cytosolic isoforms [17]. Thacker et al. described coumarin–1,2,4-oxadiazole hybrid **1C** [18], and De Luca et al. chromen-2-one **1D** as further examples combining strong dual hCA IX/XII inhibition with minimal off-target effects on hCA I/II [19]. Buran et al. also found 8-substituted coumarin **1E** [20], Bonardi et al. chromeno [4,3-c]pyrazol-4-one **1F** [21], and Kurt et al. coumarin–monoterpene **1G** [22] to all display nanomolar hCA IX/XII inhibition with negligible hCA I/II inhibition, with compound **1F** additionally reducing HT-29 colon cancer cell viability under both normoxic and hypoxic conditions. Collectively, these findings position the coumarin nucleus as a privileged, modular platform for the design of CA IX/XII–directed components in MTDLs.

Disrupting microtubule dynamics represents a second, clinically validated strategy for cancer chemotherapy [7]. Microtubules, assembled from α/β-tubulin heterodimers, are essential for mitotic spindle formation, intracellular transport, and maintenance of cell shape [23]. Microtubule-targeting agents such as taxanes and vinca alkaloids exert their cytotoxic effects by stabilizing or destabilizing microtubules, leading to mitotic arrest and apoptosis in rapidly proliferating cells [24]. However, their clinical utility is constrained by dose-limiting toxicities and multiple resistance mechanisms, including altered β-tubulin isotype expression, mutations at drug-binding sites, and enhanced efflux via ATP-binding cassette transporters [25]. These limitations have spurred the search for new tubulin-directed chemotypes, particularly small, rigid heteroaromatic systems that engage the colchicine-binding site and circumvent the cis–trans isomerization liability and poor drug-like properties of stilbene-based agents such as combretastatin A-4 [26,27].

**Figure 2:**
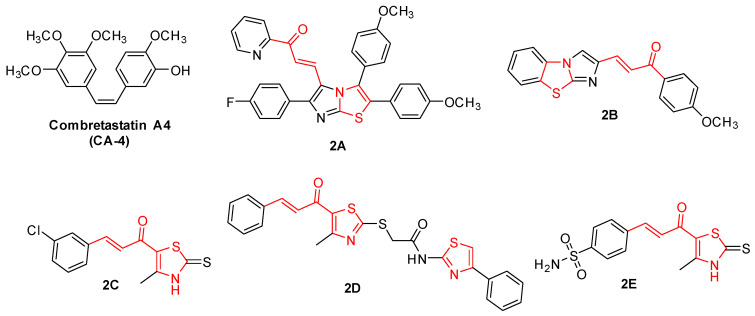
Representative thiazole–chalcone scaffolds as colchicine-site tubulin inhibitors.

Within this context, thiazole-containing chalcones have emerged as a promising pharmacophore for colchicine-site tubulin inhibition, as illustrated in Figure 2. In such scaffolds, the thiazole and pendant phenyl ring act as surrogates for the two aromatic rings of combretastatin, while the α,β-unsaturated carbonyl linker replaces the cis-alkene and provides a rigid, conjugated spacer between both termini at the colchicine site. Kamal and co-workers first validated this design with an imidazo[2,1-b]thiazole–chalcone conjugate, **2A**, a potent colchicine-site ligand that disrupted tubulin assembly and induced mitotic arrest [28]. In a follow-up study from the same group, compound **2B** was identified as a lead thiazole–chalcone hybrid with confirmed colchicine-site binding and potent tubulin polymerization inhibition [29]. Hashem and co-workers then established the thiazole–chalcone core itself as a discrete tubulin-inhibitory pharmacophore, exemplified by **2C** as a combretastatin A-4 mimetic [30]. Building on this scaffold, Al-Wahaibi and colleagues developed thiazole-2-acetamide–chalcone hybrids, with **2D** retaining strong tubulin inhibition and antiproliferative activity while sparing normal cells [31]. Khasawneh and co-workers further showed that embedding the same thiazole–chalcone motif within a sulfonamide-containing hybrid **2E** enables dual tubulin/CA IX modulation [32]. In line with these precedents, we exploit this thiazole–chalcone pharmacophore here as the colchicine-site module of a multitarget-directed design.

**Figure 3:**
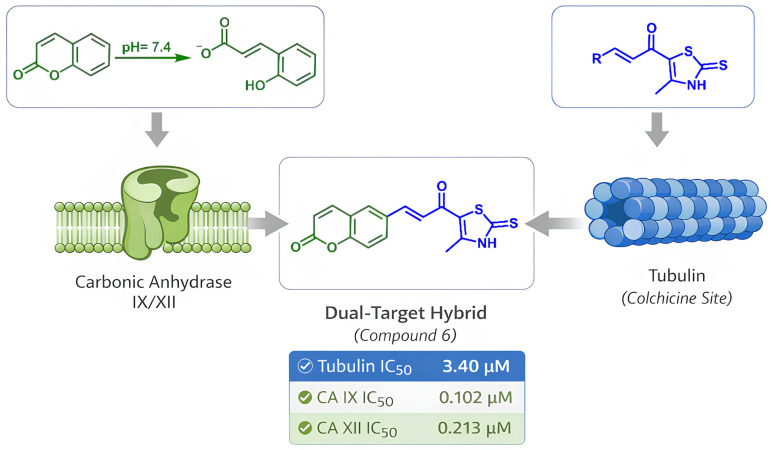
Design of the target compound **6**.

Simultaneous inhibition of CA IX/XII and tubulin offers a rational strategy to disrupt both the pH regulation and proliferative capacity of hypoxic tumors, thereby amplifying cellular stress and curbing metastatic potential [33,34]. Guided by this rationale, we sought to translate the previously discussed thiazole–chalcone lead **2C** into a dual tubulin/CA-directed scaffold by replacing its 3-chlorophenyl ring with a coumarin moiety. In this design, the thiazole–chalcone segment preserves the validated colchicine-site pharmacophore responsible for tubulin inhibition in **2C**, while the coumarin headgroup is introduced as a non–zinc-binding, entrance-directed CA warhead capable of selectively engaging CA IX/XII. The resulting coumarin–thiazole chalcone hybrid thus retains the antimitotic potential of the parent thiazole–chalcone while incorporating a CA IX/XII-addressing element, with the aim of achieving efficient dual modulation of tubulin and tumor-associated carbonic anhydrases within a compact, drug-like framework (Figure 3). To the best of our knowledge, the designed coumarin–thiazole chalcone hybrid described herein (Compound **6**) has not been previously reported.

## 2. Results and Discussion

### 2.1. Chemistry

**Scheme 1:**
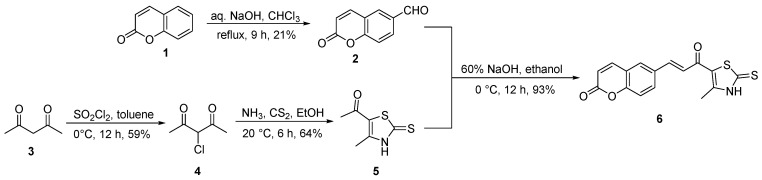
Synthesis of the target compound **6**.

The synthetic route to the coumarin–thiazole chalcone **6** is summarized in Scheme 1. Coumarin-6-carbaldehyde **2** was first obtained in good yield from commercially available coumarin via a classical Reimer–Tiemann formylation, which involves treatment of coumarin with aqueous sodium hydroxide and chloroform [35].

On the other hand, treatment of acetyl acetone **3** with sulfuryl chloride in anhydrous toluene gives the corresponding 3-chloro derivative **4** in moderate yield via selective chlorination of the activated methylene center. Subsequent reaction of **4** with carbon disulfide in ethanolic ammonia at ambient temperature led to cyclization and formation of thiazole intermediate **5** [30,36].

Finally, the target coumarin–thiazole chalcone **6** was obtained through a Claisen–Schmidt condensation between coumarin-6-carbaldehyde **2** and thiazole **5**. Equimolar amounts of **2** and **5** were stirred in ethanol in the presence of aqueous sodium hydroxide. The final compound **6** was obtained in excellent yield.

The ^1^H NMR data of compound **6** are in full agreement with the proposed coumarin–thiazole chalcone structure. A markedly downfield singlet at δ 13.59 (1H, s) corresponds to the thiazolin-2-thione NH. This highly deshielded NH resonance is consistent with the predominance of the thione tautomer under the NMR recording conditions (DMSO-*d*_6_). The conjugated enone linker is represented by two mutually coupled vinylic protons at δ 8.00 (1H, d, J = 15.0 Hz) and 7.35 (1H, d, J = 15.2 Hz), attributed to Hα and Hβ of the chalcone fragment, respectively; the large vicinal coupling constant (J ≈ 15 Hz) clearly indicates a trans-disubstituted Cα=Cβ bond and thus confirms both the E configuration and successful formation of the chalcone moiety. The remaining signals between δ 7.93 and 6.42 account for the five aromatic protons of the coumarin nucleus and display the expected pattern and coupling constants for a 6-substituted coumarin system. Finally, the singlet at δ 2.58 (3H, s), integrating for three protons, corresponds to the methyl substituent on the thiazole ring. Collectively, the chemical shifts, integrations and coupling constants unequivocally support the proposed structure of compound **6**.

### 2.2. Biological Evaluation

#### 2.2.1. Evaluation of In Vitro Antiproliferative Activity

The cytotoxic activity of compound **6** was evaluated in vitro against four human cancer cell lines—MDA-MB-231 (triple-negative breast cancer), HepG2 (hepatocellular carcinoma), A549 (lung adenocarcinoma), and HT-29 (colorectal adenocarcinoma)—as well as the non-tumorigenic breast epithelial cell line MCF-10A, using the MTT assay [37,38]. For comparative purposes, two reference agents with established anticancer profiles were included: Combretastatin A-4 (CA-4), a well-characterized tubulin polymerization inhibitor frequently used as a positive control in antiproliferative studies, and SLC-0111, a clinically advanced selective inhibitor of carbonic anhydrases IX/XII that serves as a standard reference in CA-targeted cellular assays.

**Figure 4:**
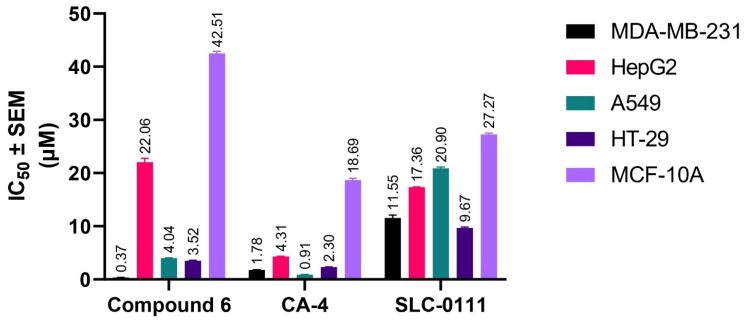
Comparative antiproliferative activity of compound **6**, CA-4, and SLC-0111 against MDA-MB-231, HepG2, A549, HT-29 cancer cell lines and non-tumorigenic MCF-10A cells, expressed as IC_50_ values (µM, mean ± SEM).

As shown by the IC_50_ values presented in Figure 4, compound **6** exhibited a cell-line–dependent activity profile across the cancer panel. The highest potency was observed in MDA-MB-231 cells, where compound **6** achieved an IC_50_ of 0.37 μM, corresponding to ~4.8-fold greater potency than CA-4 (1.78 μM) and ~31-fold greater potency than SLC-0111 (11.55 μM). This submicromolar activity indicates a strong antiproliferative effect against this aggressive TNBC phenotype. In A549 and HT-29 cells, compound **6** retained notable cytotoxicity (IC_50_ = 4.04 and 3.52 μM, respectively), demonstrating comparable or slightly reduced potency relative to CA-4 but superior activity compared with SLC-0111. HepG2 cells were comparatively less responsive to compound **6** (IC_50_ = 22.06 μM), and this model also showed relatively higher IC_50_ values for the reference agents compared with the other cell lines (e.g., CA-4 = 4.31 μM; SLC-0111 = 17.36 μM), consistent with reduced overall sensitivity in HepG2.

Evaluation of the selectivity indices (SI) in Table 1 further highlights the therapeutic potential of compound **6**; SI was calculated as IC_50_(MCF-10A)/IC_50_(cancer cell line). The SI value of 114.9 for MDA-MB-231 reflects exceptional selectivity for nonmalignant breast cells, greatly surpassing the selectivity recorded for CA-4 (10.51) and SLC-0111 (2.36). This expansive therapeutic window is rarely observed among chalcone- or coumarin-containing small molecules and underscores the strong preference of compound **6** for TNBC cells. Similarly, the SI value calculated for HT-29 cells (12.08) exceeded those of both reference agents, while for A549 cells, the SI value (10.53) surpassed that of SLC-0111 but remained slightly lower than CA-4, supporting a broader trend of favorable tumor selectivity. The lowest SI for compound **6** was observed in HepG2 (SI = 1.93), consistent with its weaker antiproliferative activity in this line.

#### 2.2.2. Effect of Compound **6** on Tubulin Polymerization

To evaluate its potential as a microtubule-disrupting agent, compound **6** was assessed for its ability to inhibit tubulin polymerization in vitro [31]. The results (Table 2) demonstrated potent activity, with an IC_50_ value of 3.40 ± 0.09 µM, which is closely comparable to the reference compound combretastatin A-4 (CA-4, IC_50_ = 3.28 ± 0.14 µM). This near-equipotent inhibitory effect underscores the strong antitubulin activity of compound **6** and supports its proposed role in disrupting microtubule dynamics.

Compared to the thiazole–phenyl chalcones reported by Hashem et al. [30], compound **6** exhibits markedly enhanced tubulin polymerization inhibition. In their study, the most active derivative, compound **2C** (Figure 2), displayed an IC_50_ of 7.78 µM—substantially higher than the 3.40 ± 0.09 µM observed for compound **6**. Although both compounds share a common thiazole–chalcone scaffold, the replacement of the terminal phenyl ring in **2C** with a coumarin moiety in compound **6** appears to significantly enhance inhibitory potency. This structural modification likely facilitates improved molecular interactions and a more favorable fit within the tubulin polymerization site, thereby improving the compound’s overall antitubulin efficacy.

#### 2.2.3. Effect of Compound **6** on Carbonic Anhydrase Isoforms (CA I, CA II, CA IX, and CA XII)

To investigate the carbonic anhydrase (CA) inhibitory potential of our designed multitarget-directed ligand, compound **6** was assessed for its in vitro activity against four physiologically and pathologically relevant CA isoforms: the cytosolic off-targets CA I and CA II, and the tumor-associated targets CA IX and CA XII [39]. Two well-characterized reference compounds were included for comparison: acetazolamide (AAZ), a prototypical pan-CA inhibitor, and SLC-0111, a clinical-stage sulfonamide currently in trials for CA IX/XII–overexpressing tumors. As shown in Table 3, compound **6** exhibited potent inhibitory activity against both tumor-associated isoforms, with IC_50_ values of 0.102 ± 0.005 µM for CA IX and 0.213 ± 0.004 µM for CA XII. Against CA IX, compound **6** displayed comparable potency to AAZ (0.105 µM) and was within ~2.1-fold of SLC-0111 (0.048 µM), highlighting its capacity to effectively inhibit this hypoxia-induced target. In the case of CA XII, compound **6** also maintained submicromolar inhibition, though it was less potent than SLC-0111 (~2.2-fold) and AAZ (~7.3-fold). Nonetheless, its activity against both tumor-associated isoforms remains well within the therapeutically desirable submicromolar range.

In contrast, compound **6** exhibited significantly weaker inhibition of the cytosolic off-target isoforms. It recorded IC_50_ values of 5.483 ± 0.12 µM for CA I and 3.142 ± 0.08 µM for CA II—approximately 15- and 21-fold weaker than AAZ, respectively, and 4.0- and 6.3-fold weaker than SLC-0111. This attenuated activity toward off-target isoforms represents a marked departure from the broad-spectrum inhibition profile of AAZ and instead parallels the more tumor-selective behavior observed with SLC-0111. Specifically, while AAZ indiscriminately inhibits all isoforms with high potency, compound **6** shows a preference for the membrane-bound, tumor-associated CA IX and XII, consistent with its intended selective profile.

This favorable selectivity was quantitatively reinforced through selectivity index (SI) calculations, defined as the ratio of IC_50_ values for off-target isoforms (CA I and II) to those for the tumor-associated isoforms (CA IX and XII). As demonstrated in Table 4, Compound **6** yielded SI values of 53.8 (CA I/CA IX), 30.8 (CA II/CA IX), 25.7 (CA I/CA XII), and 14.8 (CA II/CA XII)—all of which substantially surpassed those of AAZ (3.5, 1.5, 12.7, and 5.3, respectively) and even outperformed SLC-0111 (28.3, 10.4, 14.2, and 5.2, respectively). These findings not only validate the isoform-targeted design strategy but also highlight the potential of compound **6** to achieve therapeutic efficacy in hypoxic tumors while limiting off-target effects on physiologically critical cytosolic isoforms.

#### 2.2.4. Western Blot Analysis

**Figure 5:**
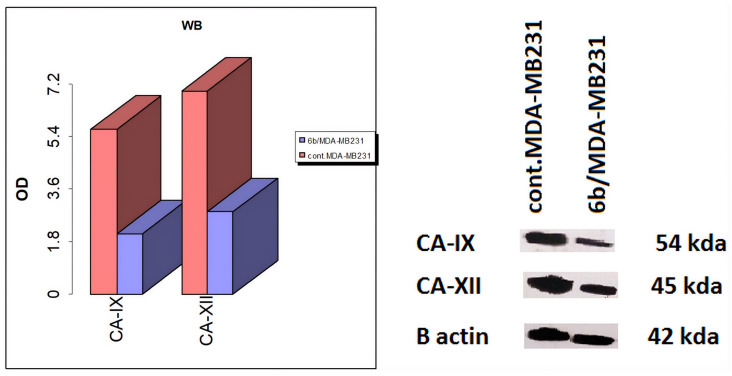
Effect of compound **6** on carbonic anhydrases IX and XII expression in the MDA-MB-231 cancer cell line.

To investigate whether compound **6** modulates tumor-associated carbonic anhydrases at the protein level, Western blot analysis was performed in MDA-MB-231 cells, and the quantitative results are summarized in Table 5, with representative immunoblots shown in Figure 5. Densitometric analysis revealed a marked reduction in both CA-IX and CA-XII expression following treatment with compound **6** compared to untreated control cells. As presented in Table 5, the normalized optical density (OD) for CA-IX decreased from 5.623 in control cells to 2.090 in compound-treated cells, while CA-XII expression declined from 6.959 to 2.820. These values correspond to an approximate 60–65% downregulation of both CA isoforms relative to the control group. Importantly, β-actin bands remained consistent across samples, confirming equal protein loading and validating the reliability of the observed differences.

The substantial suppression of CA-IX (~54 kDa) and CA-XII (~45 kDa), as visualized in Figure 5, provides direct biochemical evidence that compound **6** effectively reduces the cellular abundance of these hypoxia-associated enzymes. Given that CA-IX and CA-XII play central roles in maintaining intracellular pH balance and facilitating tumor adaptation to hypoxic microenvironments, their downregulation suggests that compound **6** interferes with cancer cell metabolic resilience.

#### 2.2.5. Cell Cycle Analysis and Apoptosis

**Figure 6:**
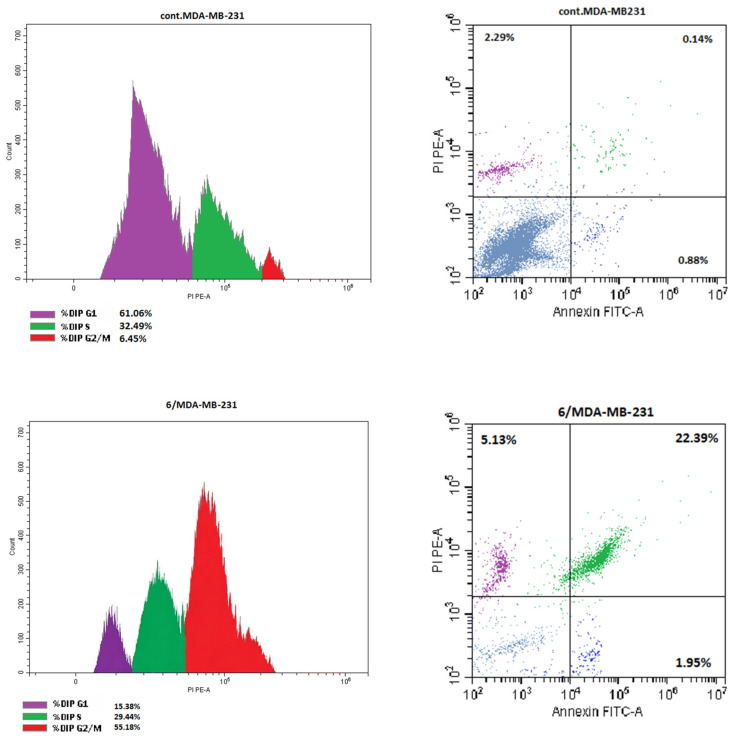
Cell cycle analysis and apoptotic detection of compound **6** in the MDA-MB-231 cancer cell line.

The effect of compound **6** on cell cycle progression and apoptosis in MDA-MB-231 cells was investigated using propidium iodide (PI)-based DNA content analysis and Annexin V–FITC/PI dual staining followed by flow cytometry. As illustrated in Figure 6, cell cycle analysis revealed a profound redistribution of cell populations upon treatment with compound **6** compared to untreated controls. Control cells predominantly resided in the G_0_/G_1_ phase (61.06%), with 32.49% in the S phase and only 6.45% in G_2_/M phase. In contrast, compound **6** treatment resulted in a dramatic accumulation of cells in the G_2_/M phase (55.18%), accompanied by a marked reduction in the G_0_/G_1_ fraction (15.38%), while the S phase population remained relatively comparable (29.44%). The more than eight-fold increase in the G_2_/M population strongly indicates mitotic arrest, consistent with the compound’s demonstrated inhibition of tubulin polymerization and docking at the colchicine-binding site. Microtubule disruption is well known to activate the mitotic checkpoint, leading to G_2_/M phase accumulation and subsequent apoptotic signaling, thereby supporting the mechanistic basis of compound **6**–mediated cytotoxicity.

In parallel, Annexin V–FITC/PI dual staining analysis (Figure 6) demonstrated a significant induction of apoptosis in compound **6**–treated cells. Total apoptotic cells increased markedly from 3.31% in the control group to 29.47% following treatment (Table 6). Notably, late apoptosis constituted the predominant fraction (22.39%), while early apoptosis accounted for 1.95%, and necrosis showed only a moderate increase to 5.13%, compared to 0.88% early apoptosis, 0.14% late apoptosis, and 2.29% necrosis in control cells. The predominance of late apoptotic cells suggests progression beyond the early apoptotic stage and supports activation of a regulated apoptotic process rather than nonspecific cytotoxic necrosis. Collectively, the results shown in Figure 4 indicate that compound **6** induces pronounced G_2_/M phase arrest followed by substantial apoptotic cell death in MDA-MB-231 cells, providing mechanistic support for its antimitotic and cytotoxic activity.

#### 2.2.6. Effect of Compound **6** on Mitochondrial Apoptosis Markers (Bax, Bcl-2, and Cytochrome C)

To further elucidate the cellular mechanism underlying the pronounced antiproliferative effect of compound **6** in MDA-MB-231 cells, key regulators of the intrinsic (mitochondria-dependent) apoptotic pathway were evaluated. Specifically, the expression levels of Bax, Bcl-2, and cytochrome c were quantified and compared to DMSO-treated controls (Table 7) [40]. Results revealed that compound **6** induced a coordinated pro-apoptotic response, consistent with mitochondrial pathway activation.

Treatment with compound **6** resulted in a significant upregulation of the pro-apoptotic protein Bax, increasing from 102.09 ± 2.27 pg/µL in the control group to 505.28 ± 5.46 pg/µL—a 4.95-fold elevation. Given Bax’s critical role in promoting mitochondrial outer membrane permeabilization (MOMP), this increase suggests amplification of upstream death signaling and facilitation of mitochondrial destabilization.

Simultaneously, expression of Bcl-2, a key anti-apoptotic protein that antagonizes BAX/BAK-mediated MOMP, was markedly downregulated following exposure to compound **6**. Its concentration dropped from 17.88 ± 0.11 pg/µL in the control to 5.72 ± 0.08 pg/µL, corresponding to a 0.320-fold relative level. The opposing regulation of Bax and Bcl-2 markedly shifted the apoptotic balance, with the BAX/Bcl-2 ratio rising from ~5.71 in the control to ~88.34 after treatment—a ~15.5-fold increase. This dramatic shift supports mitochondrial commitment toward apoptosis and aligns with canonical apoptotic regulatory mechanisms.

In accordance with these upstream changes, compound **6** also triggered a pronounced increase in cytochrome c levels, which rose from 0.075 ± 0.005 ng/µL in control cells to 0.680 ± 0.030 ng/µL following treatment—a 9.07-fold elevation. This substantial increase in cytochrome c levels is strongly indicative of mitochondrial membrane permeabilization and downstream apoptotic signaling, aligning with the observed modulation of Bax and Bcl-2.

#### 2.2.7. Effect of Compound **6** on Caspase-9 and Caspase-3 Expression Levels

To further confirm apoptosis induction, the effects of compound **6** on caspacse-9 (initiator caspase in the intrinsic pathway) and caspase-3 (major executioner caspase) were measured in MDA-MB-231 cells and compared with the DMSO control [41]. As shown in Table 8, compound **6** markedly increased both markers, supporting activation of a caspase-dependent apoptotic process.

Compound **6** caused a strong increase in caspase-9, rising from 5.625 ± 0.275 ng/µL in the control to 58.57 ± 1.44 ng/µL, corresponding to a 10.4-fold elevation. Since caspase-9 is typically activated after mitochondrial signaling and cytochrome c release, this result is consistent with prominent engagement of the intrinsic (mitochondrial) apoptotic pathway. In the same direction, caspase-3 increased from 43.50 ± 1.5 pg/µL (DMSO) to 456.5 ± 7.5 pg/µL, giving a 10.49-fold rise. This substantial elevation indicates efficient progression to the execution stage of apoptosis, where caspase-3 mediates widespread cleavage of cellular targets and drives apoptotic cell death.

#### 2.2.8. HUVEC Wound-Healing (Scratch) Migration Assay

Endothelial cell migration is a key step in angiogenic sprouting; therefore, we evaluated the effect of compound **6** on collective endothelial motility using a standard wound-healing (scratch) assay, in which a linear “scratch” is generated in a confluent monolayer and closure is quantified over time. Under these conditions, compound **6** produced a clear anti-migratory phenotype in HUVECs, reducing wound closure at 72 h to 65.185 ± 2.1% compared with 94.815 ± 3.05% in the control group (Table 9). This corresponds to an absolute decrease of ~29.6 percentage points (≈31% relative reduction vs. control), indicating that compound **6** substantially delays endothelial gap closure rather than permitting near-complete re-epithelialization as observed in untreated cells.

**Figure 7:**
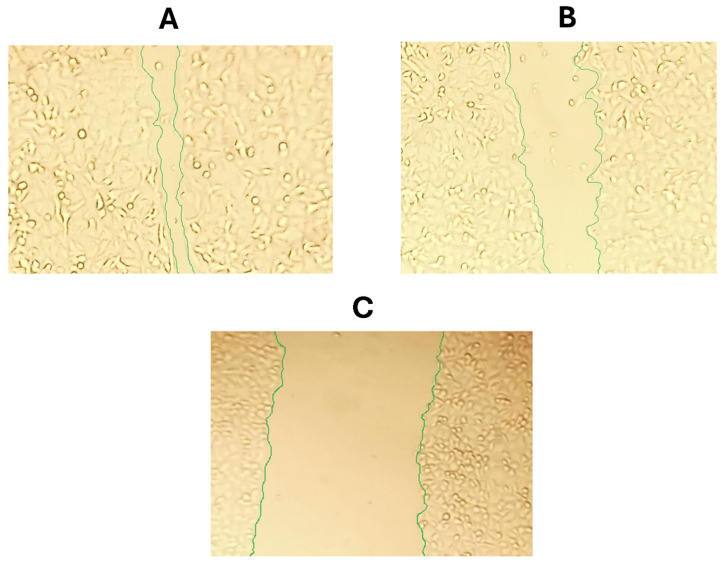
Representative phase-contrast micrographs of HUVEC scratch wound-healing assay. (**A**) Vehicle-treated control, (**B**) compound **6**–treated group, and (**C**) baseline immediately after scratch (t = 0). Green lines delineate the wound margins.

The microscopy images (Figure 7) support the quantitative outcome, showing a visibly wider residual wound area in the compound-treated condition compared with the control, whereas the initial scratch is shown in panel C. Together with the quantitative closure data (Table 9), these findings are consistent with inhibition of endothelial migration, a functional hallmark associated with anti-angiogenic activity.

#### 2.2.9. Inhibition of VEGFR-2 Phosphorylation in HUVEC Cells

**Figure 8:**
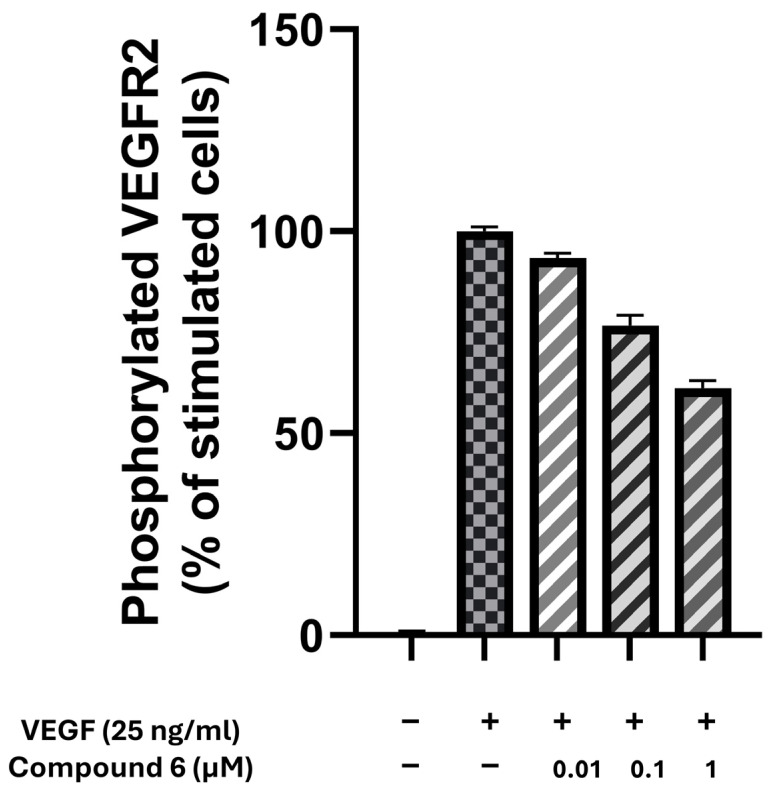
Effect of compound **6** on VEGF-stimulated VEGFR-2 (Tyr1175) phosphorylation in HUVEC cells.

VEGFR-2 is the principal receptor mediating VEGF-driven signaling in endothelial cells, and phosphorylation at Tyr1175 is a key activation node that recruits downstream effectors (e.g., PI3K/PLCγ-associated signaling), ultimately supporting endothelial proliferation, chemotaxis/sprouting, and angiogenesis. In the present study, VEGF-stimulated HUVECs were used to evaluate whether compound **6** can attenuate VEGFR-2 activation at Tyr1175 using the rapid PathScan RP Phospho-VEGFR-2 (Tyr1175) sandwich ELISA, in which signal intensity is proportional to endogenous phospho-VEGFR-2 levels. Compound **6** produced a clear concentration-dependent suppression of VEGFR-2 (Tyr1175) phosphorylation (Table 10), yielding 6.73 ± 1.28%, 23.29 ± 2.47%, and 38.84 ± 1.93% inhibition at 0.01, 0.1, and 1 µM, respectively. Consistent with these quantitative data, the accompanying bar plot (Figure 8) shows robust induction of phospho-VEGFR-2 by VEGF (set to 100%), while compound **6** progressively reduced the VEGF-driven signal to ~93%, ~77%, and ~61% of stimulated control across the same concentration range, supporting partial but measurable blockade of receptor activation. Collectively, these results provide mechanistic support for the anti-angiogenic activity of compound **6** in HUVEC cells by demonstrating that it can suppress VEGF/VEGFR-2 signaling at Tyr1175—an event directly linked to endothelial activation programs relevant to migration and neovessel formation.

#### 2.2.10. In Vitro Human Liver Microsomal Stability

To complement the in vitro pharmacological profile of compound **6** and provide an estimate of its susceptibility to oxidative metabolism, the metabolic stability of compound **6** was evaluated in pooled human liver microsomes (HLM) in the presence of NADPH. Depletion kinetics were monitored by LC–MS/MS using diazepam as an internal standard, and the log-transformed peak-area ratios showed an approximately linear decline over time, supporting the use of a first-order depletion model to derive kinetic parameters.

Across the examined starting concentrations (0.8–20 µM), compound **6** exhibited concentration-dependent microsomal stability, with a progressive increase in half-life and reduction in apparent intrinsic clearance as the initial concentration increased. Specifically, the half-life increased from 0.571 ± 0.028 h at 0.8 µM to 0.883 ± 0.067 h at 4 µM and 1.421 ± 0.079 h at 20 µM, accompanied by a decrease in CLint, in vitro from 40.53 ± 1.96 to 26.22 ± 2.00 and 16.28 ± 0.91 µL/min/mg, respectively (Table 11). Consistently, the remaining parent signal at 60 min increased from ~30.0 ± 1.7% (0.8 µM) to ~44.9 ± 4.4% (4 µM) and ~63.3 ± 0.7% (20 µM). This trend is compatible with an apparent capacity-limited (saturable) metabolic component within the tested range, whereby higher substrate levels reduce the fractional depletion rate under fixed microsomal protein conditions. While microsomal assays provide an initial estimate of metabolic liability rather than a full pharmacokinetic characterization, these data suggest that compound **6** has measurable stability in human hepatic microsomes, with an improved apparent stability at higher substrate concentrations.

### 2.3. In Silico Studies

#### 2.3.1. Molecular Docking

##### Docking Into Carbonic Anhydrase IX and XII

Given that coumarins exert their carbonic anhydrase (CA) inhibitory activity through an enzyme-mediated hydrolysis mechanism, which converts the parent lactone into a 2-hydroxycinnamic acid derivative, molecular docking investigations were conducted using the hydrolyzed form of compound **6** [14,42]. This strategy aligns with crystallographic and mechanistic reports indicating that the active inhibitory species in CA–coumarin complexes binds at the entrance of the catalytic cleft, rather than directly coordinating the catalytic zinc ion, as observed with sulfonamide-based CA inhibitors. Both E and Z isomers of the hydrolyzed compound were first independently docked into the hCA IX active site to elucidate the preferred orientation and key binding interactions underpinning its potent and selective inhibition. The E isomer exhibited a binding affinity of −7.6 kcal/mol, while the Z isomer showed −6.8 kcal/mol, indicating a clear energetic preference for the E configuration in CA IX.

**Figure 9:**
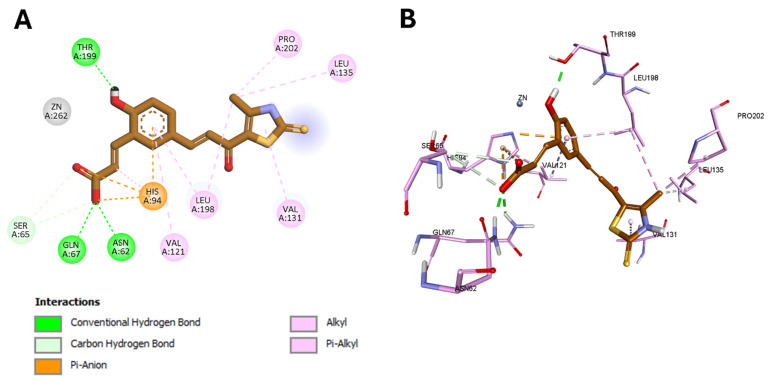
Docking of the hydrolyzed E-isomer of compound **6** into hCA IX: (**A**) 2D interaction diagram and (**B**) 3D binding pose at the entrance of the catalytic site.

The docking results of the E isomer revealed a well-defined binding pose situated at the rim of the active site, stabilized by an extensive polar and hydrophobic interaction network (Figure 9). The carboxylate moiety engaged in classical hydrogen bonds with Gln67 and Asn62, alongside carbon–hydrogen bonds with Ser65, forming a robust polar anchoring triad. Additionally, the carboxylate contributed to a π-anion electrostatic interaction with His94, which further reinforced its positioning at the cleft entrance. The phenolic hydroxyl group served as a critical hydrogen bond donor to Thr199, a residue frequently implicated in the recognition of entrance-binding CA inhibitors.

Hydrophobic interactions also played a substantial role in stabilizing the E-isomer orientation. The benzene ring of the cinnamic acid segment formed π-alkyl interactions with Val121 and Leu198, while simultaneously maintaining electrostatic engagement with His94 through its aromatic system. The methyl substituent on the thiazole ring exhibited alkyl contacts with Leu198, Pro202, and Leu135, effectively anchoring the chalcone arm. The thiazole ring itself participated in a π-alkyl interaction with Val131, facilitating additional hydrophobic complementarity within the entrance region. This collective arrangement of contacts is consistent with the entrance-binding behavior observed in structurally related coumarin-based inhibitors, which have been structurally characterized as occupying analogous entrance-rim positions with minimal direct zinc coordination.

**Figure 10:**
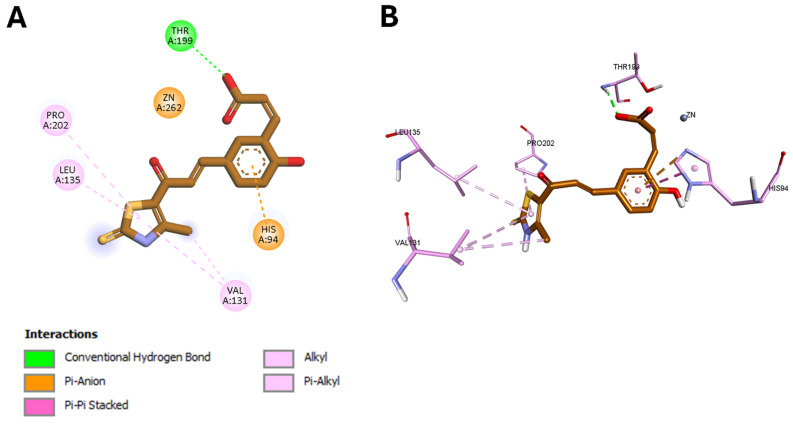
Docking of the hydrolyzed Z-isomer of compound **6** into hCA IX: (**A**) 2D interaction diagram and (**B**) 3D binding pose at the entrance of the catalytic site.

In comparison, the Z isomer adopted a similar entrance-oriented configuration but exhibited a more limited interaction profile (Figure 10). The carboxylate group formed a single classical hydrogen bond with Thr199, in agreement with its role as a conserved polar anchor. The benzene ring preserved the π-anion electrostatic interaction with His94, indicating consistent entrance alignment. However, unlike the E isomer, the Z configuration lacked hydrogen bond interactions with Gln67, Asn62, or Ser65, suggesting a reduction in polar stabilization. Its lower binding affinity (−6.8 kcal/mol) is consistent with this diminished polar anchoring network.

Hydrophobic interactions were retained, with the methyl group on the thiazole forming an alkyl contact with Val131, while the thiazole ring displayed π-alkyl interactions with Val131, Pro202, and Leu135. Although this hydrophobic complementarity supports residence at the cleft entrance, the absence of the E isomer’s triad of hydrogen bonds may result in diminished affinity or reduced entropic favorability.

To further examine the effect of thiazole tautomerism on carbonic anhydrase binding, the hydrolyzed E-isomer of the thiol tautomer of compound **6** was additionally docked into hCA IX. The predicted binding affinity was −7.5 kcal/mol, indicating a binding mode comparable to that of the thione-based model. The hydrolyzed coumarin carboxylate contributed a classical hydrogen bond with Asn62, a carbon–hydrogen bond with Ser65, and a π-anion electrostatic interaction with His94. The hydrolyzed coumarin OH formed a classical hydrogen bond with Thr199. The coumarin benzene ring showed π-anion interaction with His94 and hydrophobic π-alkyl contacts with Leu198 and Val121, while the thiazole ring and its methyl substituent contributed hydrophobic contacts with Val131, Leu91, and Val131, respectively. These interactions are shown in Figure S3 (Supplementary Materials).

**Figure 11:**
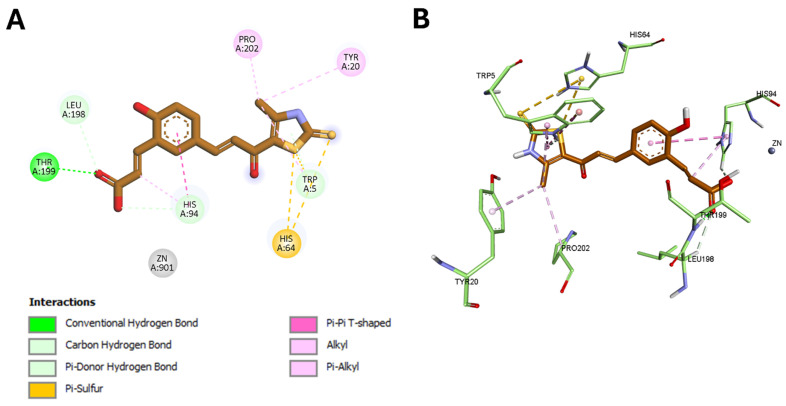
Docking of the hydrolyzed E-isomer of compound **6** into hCA XII: (**A**) 2D interaction diagram and (**B**) 3D binding pose at the entrance of the catalytic site.

**Figure 12:**
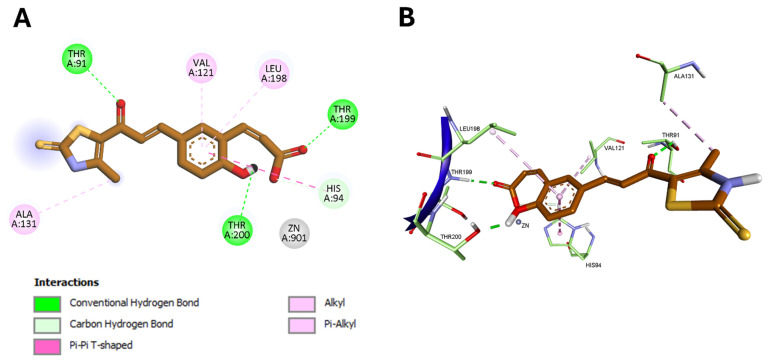
Docking of the hydrolyzed Z-isomer of compound **6** into hCA XII: (**A**) 2D interaction diagram and (**B**) 3D binding pose at the entrance of the catalytic site.

The docking of hydrolyzed compound **6** into hCA XII also revealed entrance-directed binding for both isomers, albeit with reduced binding affinity compared to hCA IX (Figure 11 and Figure 12). The E and Z isomers yielded docking scores of −6.9 and −6.4 kcal/mol, respectively, indicating weaker energetic stabilization within the CA XII entrance site. The E isomer formed a classical hydrogen bond between the carboxylate and Thr199, alongside carbon–hydrogen bonds with His94 and Leu198, collectively anchoring the compound at the rim. The aromatic core was stabilized by a T-shaped π–π interaction with His94, while the thiazole–chalcone segment extended into a hydrophobic subpocket. The thiazole methyl group interacted with Pro202 and formed π-alkyl contacts with Tyr20 and Trp5. Notably, the thiazole and thione moieties established π-sulfur interactions with Trp5 and His64, and a π-donor hydrogen bond with Trp5, adding rare but chemically meaningful interaction types that may enhance selectivity and binding persistence at the entrance site.

The Z isomer, while similarly anchored to the rim, exhibited a more polar-dominated interaction profile. The carboxylate retained a hydrogen bond with Thr199 and a carbon–hydrogen bond with His94, and the phenolic OH engaged Thr200, further strengthening the polar anchoring. The chalcone carbonyl additionally formed a classical hydrogen bond with Thr91, suggesting a stabilizing network along the ligand backbone. Hydrophobic interactions were preserved through π-alkyl contacts with Val121 and Leu198, and an alkyl contact between the thiazole methyl and Ala131. A T-shaped π–π interaction with His94 was again observed, confirming the aromatic ring’s consistent contribution to rim recognition. Despite these interactions, the reduced interaction density and lower docking score (−6.4 kcal/mol) distinguish CA XII binding from the more favorable and tightly anchored CA IX complex.

The hydrolyzed E-isomer of the thiol tautomer was also docked into hCA XII to assess tautomer-dependent changes in binding. The predicted binding affinity was −6.5 kcal/mol. The hydrolyzed coumarin carboxylate formed a classical hydrogen bond with Thr199 and a carbon–hydrogen bond with Leu198, while the hydrolyzed coumarin OH showed a carbon–hydrogen bond with His94. The coumarin benzene ring displayed a π–π T-shaped interaction with His94. In addition, the thiazole ring and methyl substituent contributed hydrophobic contacts with Val131, Leu91, and His64, and the thiazole ring also showed a non-classical π-donor hydrogen bond and π–π T-shaped interactions with Trp5. Overall, the thiol tautomer preserved an entrance-directed binding mode in hCA XII, although with a less favorable predicted affinity than the corresponding thione-based pose. These interactions are shown in Figure S4 (Supplementary Materials).

##### Docking Into Colchicine Binding Site of Tubulin

Following the carbonic anhydrase docking results, molecular docking was performed to investigate the binding mode of compound **6** at the colchicine-binding site of tubulin, providing a mechanistic rationale for its experimentally confirmed tubulin polymerization inhibition [27]. The molecular scaffold of compound **6** retains the validated thiazole–chalcone pharmacophore, previously established as a colchicine-site ligand, while introducing a coumarin moiety in place of the typical aryl group, aiming to extend the interaction network without compromising key binding interactions.

Docking studies revealed a well-defined pose that closely aligns with the binding orientation reported for phenyl-substituted thiazole chalcones, such as compound **2C** from the work of Hashem et al. In their study, compound **2C** engaged the colchicine-binding domain primarily through hydrogen bonding with Cys241, π-interactions, and hydrophobic anchoring via the thiazole ring. Compound **6** preserves these critical interactions while demonstrating an expanded binding profile due to its extended aromatic framework. This binding configuration was associated with a calculated docking score of −8.1 kcal/mol, indicating a strong and energetically favorable interaction with the colchicine site.

**Figure 13:**
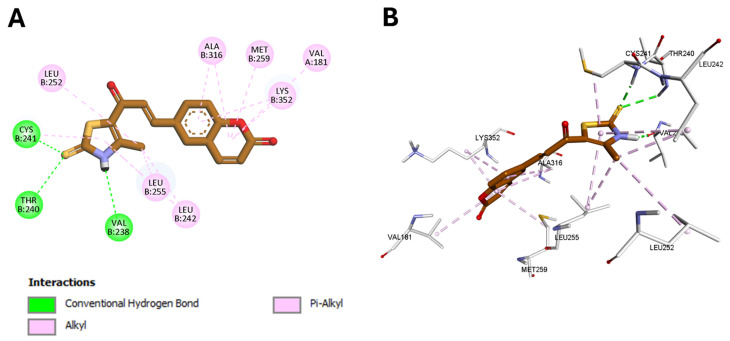
Docking of compound **6** into the colchicine-binding site of tubulin: (**A**) 2D interaction diagram and (**B**) 3D binding pose illustrating stabilization at the β-tubulin interface.

The thione group of compound **6** forms classical hydrogen bonds with Cys241 and Thr240, anchoring the molecule at the β-tubulin interface. Additionally, the thiazole NH group donates a hydrogen bond to Val238, further stabilizing the interaction with the protein backbone. The thiazole–thione moiety is further stabilized by π-alkyl interactions with Cys241, Leu242, and Leu255, while the methyl substituent contributes additional hydrophobic contacts with Leu242 and Leu255. These interactions recapitulate those observed in reference chalcones, which rely on hydrophobic interactions with Leu255, Ala316, and nearby residues for spatial orientation and affinity. A distinguishing feature of compound **6** is the presence of the coumarin moiety, which replaces the phenyl group found in compound **2C**. In this context, this fused benzopyrone system participates in π-alkyl interactions with a broader array of hydrophobic residues, including Ala316, Met259, Val181, and Lys352, resulting in deeper insertion within the binding pocket and increased interaction surface. This broader hydrophobic engagement likely contributes to the slightly enhanced potency observed for compound **6** compared to compound **2C** (3.40 µM vs. 7.78 µM). These interactions are illustrated in Figure 13.

To investigate tautomeric effects in the tubulin binding site, the intact E-isomer of the thiol tautomer of compound **6** was docked into the colchicine-binding pocket. The predicted binding affinity was −8.0 kcal/mol. The coumarin ring formed hydrophobic π-alkyl interactions with Met259, Val181, Lys352, and Ala316. The thiazole methyl group formed a hydrophobic alkyl interaction with Leu242, while the thiazole ring displayed hydrophobic π-alkyl interactions with Leu242 and Cys241, and a π-sigma interaction with Leu255. Notably, the thiol (SH) group formed a classical hydrogen bond with Tyr202. The intact thiol tautomer therefore retained a productive binding pose in the colchicine site, with a comparable predicted affinity to the corresponding thione-based model. These interactions are shown in Figure S5 (Supplementary Materials).

#### 2.3.2. In Silico ADMET Prediction

To complement the target-engagement insights from docking and to gauge developability at an early stage, compound **6** was further profiled in silico using ADMETlab 3.0 online tool [43]. Overall, the predicted physicochemical space is compatible with a drug-like small molecule and is consistent with the robust cellular and enzymatic activity observed for this dual-acting scaffold, while also highlighting specific parameters that can be tuned during lead optimization.

**Figure 14:**
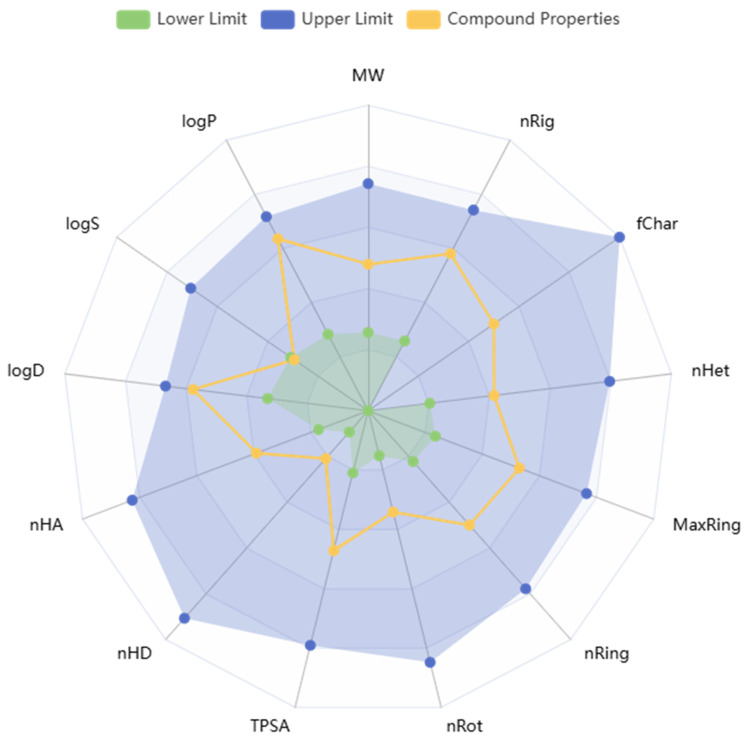
Bioavailability radar of compound **6**, illustrating key physicochemical properties relative to the optimal oral drug-likeness space, with compound values shown against defined lower and upper limits.

From a physicochemical and drug-likeness standpoint, compound **6** displays a favorable balance between polarity, hydrogen-bonding capacity, and molecular size. The predicted molecular weight (MW = 329.02) and topological polar surface area (TPSA = 63.07 Å^2^) fall within ranges typically associated with efficient passive permeability and oral drug-likeness, while maintaining sufficient polarity to support productive polar anchoring at protein binding sites. The molecule is predicted to be neutral (formal charge = 0), with restrained hydrogen-bonding features (HBD = 1; HBA = 4) and limited conformational flexibility (nRot = 3; flexibility index = 0.15), which together can reduce entropic penalties upon binding and support defined bioactive conformations. Lipophilicity metrics are moderate (logP = 2.437; logD = 2.461), consistent with the experimentally observed potency against intracellular and membrane-associated targets. In agreement, the bioavailability radar shown in Figure 14 indicates that the compound’s key descriptors largely reside between the lower and upper boundaries across multiple axes (MW, TPSA, HBD/HBA, rigidity/flexibility, ring counts, and lipophilicity), supporting an overall drug-like profile. The principal physicochemical limitation predicted is aqueous solubility (logS = −4.154), suggesting that dissolution could be a rate-limiting step for oral exposure in the absence of formulation strategies or modest polarity/ionization tuning.

These features are further reinforced by the medicinal chemistry filters: compound **6** shows no Lipinski violations (Lipinski rule: 0), and it is predicted to be free of major substructure-based liabilities typically associated with false positives or nonspecific activity, including no PAINS alerts and no chelator alerts. In practical terms, this supports the interpretation that the observed bioactivity is more likely to arise from genuine target engagement rather than overt pan-assay interference. In addition, the predicted synthetic accessibility score (Synth = 2.0) is favorable, aligning with the experimentally straightforward synthesis and supporting the feasibility of iterative analog generation for structure–property optimization.

In the absorption/permeability panel, the model predicts permissive properties for membrane passage. The Caco-2 permeability (−5.133) is close to the optimal region used by the model, and the PAMPA value (0.235) also supports passive diffusion. Importantly, the compound is predicted to show low P-gp liability, being neither a strong P-gp substrate (0.0) nor a prominent P-gp inhibitor (0.107), which is advantageous for maintaining intracellular exposure and mitigating efflux-driven resistance—particularly relevant for tubulin-directed chemotypes. In the radar plot, this absorption-friendly behavior is consistent with the moderate lipophilicity and TPSA values, as the compound does not exceed the upper-limit envelope on the polarity/lipophilicity axes.

For distribution, the predictions suggest a profile compatible with peripheral/tumor exposure and a reduced likelihood of central nervous system penetration. The compound is classified as BBB-negative (0.0), which is generally desirable for an anticancer lead to reduce CNS-related adverse effects unless brain delivery is explicitly intended. The model predicts high plasma protein binding (PPB = 98.64%), a common feature among aromatic, moderately lipophilic scaffolds; while high PPB can reduce free fraction, it may also contribute to distribution kinetics and apparent exposure. The predicted Vdss (−0.088) indicates a distribution behavior that is not excessively tissue-sequestering by the model’s criteria. Collectively, the distribution readouts align with the radar’s indication of controlled size, polarity, and lipophilicity.

Finally, the safety-relevant predictions include a particularly encouraging signal regarding cardiac liability: the probability of hERG blockade is low (0.041), which is a favorable early indicator for a tubulin-active scaffold class where ion-channel liabilities can be problematic. In addition, the model predicts no skin sensitization (0.265), supporting a generally acceptable early safety profile on that endpoint. Taken together with the overall drug-like physicochemical space captured by the radar, these findings strengthen the case that compound **6** combines potent dual pharmacology with broadly reasonable ADMET properties, which makes it a promising lead compound.

## 3. Conclusions

In summary, a structurally novel coumarin–thiazole chalcone hybrid was successfully designed and synthesized as a multitarget anticancer agent capable of simultaneously modulating tumor metabolism, mitotic progression, and angiogenic signaling. Compound **6** exhibited potent and selective inhibition of tumor-associated carbonic anhydrases IX and XII alongside strong suppression of tubulin polymerization, translating into pronounced antiproliferative activity with exceptional selectivity toward non-tumorigenic cells. Mechanistic studies demonstrated G_2_/M phase arrest and apoptosis induction mediated through mitochondrial pathway activation and caspase engagement, while Western blot analysis confirmed significant downregulation of CA-IX and CA-XII protein expression. Furthermore, the compound displayed anti-angiogenic potential by inhibiting endothelial migration and attenuating VEGFR-2 phosphorylation in HUVEC cells. Human liver microsomal experiments revealed measurable metabolic stability with reduced intrinsic clearance at higher concentrations. Together with supportive molecular docking and favorable ADMET predictions, these findings establish compound **6** as a promising multitarget scaffold integrating antimitotic, metabolic, and anti-angiogenic mechanisms. A limitation of the present study is that the biological evaluation was conducted on a single rationally designed lead (compound **6**), which limits broader SAR-based generalization. Expansion to a congeneric derivative series with full biological profiling will be pursued in future work to strengthen structure–activity conclusions.

## 4. Experimental

### 4.1. Chemistry

**General Details:** Refer to Supplementary Materials.

**General procedure for the synthesis of target compound 6.**

A mixture of coumarin-6-carbaldehyde 2 (1.0 mmol, 174 mg) and thiazole intermediate 5 (1.0 mmol, 173 mg) was dissolved in absolute ethanol and stirred at room temperature. Then, a 60% aqueous NaOH solution (3.5 mmol; 140 mg NaOH) was added dropwise with continuous stirring, and the reaction mixture was stirred at room temperature for 12 h. After completion, the solvent was removed under reduced pressure, and the residue was re-dissolved in distilled water. The resulting solution was acidified with dilute acetic acid, and the formed yellow precipitate was filtered off, washed thoroughly with distilled water, dried, and recrystallized from acetonitrile to afford compound **6** as a yellow solid.

**(*E*)-6-(3-(2-mercapto-4-methylthiazol-5-yl)-3-oxoprop-1-en-1-yl)-2H-chromen-2-one.**

Yellow powder (acetonitrile) in (260 mg, 79% yield), m.p: 218–220 °C; IR spectra (KBr cm^−1^): 3174 (N–H str of thiazole), 3012 (C–H str of Ar), 2893 (C–H str of CH_3_), 1739 (C=O str of coumarin), 1618 (C=O str of chalcone), 1266 (C=S str of thiazole); ^1^H NMR (400 MHz, DMSO-*d_6_*) δ (ppm): 13.59 (s, 1H, thiazole NH), 8.08 (d, *J* = 15.0 Hz, 1H, Chalcone CH), 7.82 (d, *J* = 1.4 Hz, 1H, Coumarin CH), 7.76 (d, *J* = 10.8 Hz, 1H, Coumarin CH), 7.73 (dd, *J* = 7.5, 1.4 Hz, 1H, Coumarin CH), 7.36 (d, *J* = 7.5 Hz, 1H, Coumarin CH), 7.20 (d, *J* = 15.0 Hz, 1H, Chalcone CH), 6.43 (d, *J* = 10.8 Hz, 1H, Coumarin CH), 2.58 (s, 3H, CH_3_); ^13^C NMR (100 MHz, DMSO-*d_6_*) δ (ppm): 189.41, 179.36, 162.43, 155.78, 149.72, 147.60, 141.26, 138.61, 133.55, 129.31, 126.14, 125.08, 122.44, 118.20, 113.96, 15.35. Anal. Calcd. For C_16_H_11_NO_3_S_2_ (329.39): C, 58.34; H, 3.37; N, 4.25. Found: C, 58.21; H, 3.45; N, 4.12.

### 4.2. Biological Evaluation

#### 4.2.1. Antiproliferative Activity

The antiproliferative activity of compound **6** was assessed using the MTT assay in MDA-MB-231, HepG2, A549, HT-29, and non-tumorigenic MCF-10A cells. All cell lines were obtained from the Vacsera Cell Culture Library, Tissue Culture Unit, Cairo, Egypt, with ATCC certification. After 72 h of treatment with varying concentrations of compound **6** or reference drugs (CA-4, SLC-0111), cell viability was determined based on the reduction in MTT to formazan by metabolically active cells. Absorbance was measured at 570 nm with 690 nm as a reference, and IC_50_ values were calculated from the resulting dose–response curves. Full experimental details are provided in Supplementary Materials.

#### 4.2.2. Tubulin Polymerization Inhibition

The effect of compound **6** on microtubule assembly was evaluated using a tubulin polymerization assay kit (BK011P, Cytoskeleton Inc., Denver, CO, USA) according to the manufacturer’s instructions. Tubulin polymerization was monitored spectrophotometrically at 340 nm at 37 °C in the presence of compound **6** or the reference inhibitor CA-4, and inhibitory effects were determined relative to the DMSO control. Detailed assay conditions and analysis steps are described in Supplementary Materials.

#### 4.2.3. Carbonic Anhydrase Inhibition

The ability of compound **6** to inhibit carbonic anhydrase activity was assessed using a colorimetric ELISA-based protocol provided by the manufacturer. The enzyme was preincubated with varying concentrations of the compound prior to substrate addition. Absorbance was measured at 405 nm, and the percentage inhibition was calculated relative to a DMSO-treated control group. IC_50_ values were derived from the dose–response data. Further assay details and procedural steps are provided in Supplementary Materials.

#### 4.2.4. Western Blotting

Protein expression levels of carbonic anhydrase IX (CAIX) and carbonic anhydrase XII (CAXII) were evaluated by Western blotting. Following compound treatment, cells were lysed in RIPA buffer containing protease inhibitors, and equal amounts of protein were separated by SDS-PAGE and transferred to PVDF membranes. Membranes were incubated with primary antibodies against CAIX and CAXII, followed by HRP-conjugated secondary antibodies and chemiluminescent detection. β-Actin was used as a loading control, and band intensities were quantified by densitometry and normalized to β-actin levels. A detailed experimental protocol is provided in Supplementary Materials.

#### 4.2.5. Cell Cycle Analysis

The effect of compound **6** on cell cycle progression in MDA-MB-231 cells was assessed by flow cytometry following propidium iodide DNA staining. After treatment, cells were harvested, fixed in cold ethanol, and stained with propidium iodide/RNase solution prior to analysis. DNA content histograms were used to determine the distribution of cells across G0/G1, S, and G2/M phases. Flow cytometric acquisition was performed using 488 nm excitation with PI fluorescence detected in the FL2 channel, and data were processed using FlowJo software (v10.8.1, BD Life Sciences). Further experimental details are described in Supplementary Materials.

#### 4.2.6. Apoptosis Assay

Apoptosis induction by compound **6** in MDA-MB-231 cells was quantified using Annexin V-FITC/propidium iodide dual staining followed by flow cytometry. After treatment, cells were harvested and incubated with Annexin V-FITC and propidium iodide in binding buffer prior to analysis. Flow cytometric acquisition enabled discrimination of viable, early apoptotic, late apoptotic, and necrotic cell populations based on fluorescence signals. Additional experimental details are provided in Supplementary Materials.

#### 4.2.7. Effect on Apoptotic Markers Bax, Bcl-2, and Cytochrome C

To assess mitochondrial apoptotic signaling, the protein levels of Bax, Bcl-2, and cytochrome c were quantified in MDA-MB-231 cells using sandwich ELISA kits according to the manufacturers’ protocols (DRG, Elabscience, and BT-Laboratory, respectively). Absorbance values were measured using a microplate reader, and concentrations were interpolated from standard curves. Additional experimental details are available in Supplementary Materials.

#### 4.2.8. Effect on Caspases 3 and 9

The activities of caspase-3 and caspase-9 in MDA-MB-231 cells treated with compound **6** were quantified using Invitrogen™ Human Active Caspase-3 ELISA Kit (Thermo Fisher Scientific, Waltham, MA, USA; catalog no. BMS2018INST) and Invitrogen™ Human Caspase-9 Instant ELISA Kit (Thermo Fisher Scientific, Waltham, MA, USA; catalog no. BMS2051INST), respectively, following the manufacturer’s protocols. Samples were prepared from treated and control cells, and optical densities were measured using a microplate reader. Detailed methodology is provided in Supplementary Materials.

#### 4.2.9. Wound Healing (Scratch) Assay on HUVEC

HUVEC cells were seeded in multiwell plates and cultured until a confluent monolayer was formed. A linear scratch was generated using a sterile pipette tip, detached cells were removed by gentle washing, and cells were then incubated in fresh medium containing the test compound (vehicle-treated wells served as controls). Images were captured immediately after scratching (0 h) and at the indicated time points using phase-contrast microscopy at fixed positions, and wound closure was quantified by image analysis as percent closure relative to the initial wound width/area. Details are provided in Supplementary Materials.

#### 4.2.10. Inhibition of VEGFR-2 Phosphorylation in HUVEC Cells

Phosphorylated VEGFR-2 (Tyr1175) levels were quantified using the PathScan^®^ RP Phospho-VEGFR-2 (Tyr1175) Sandwich ELISA Kit (#7335, Cell Signaling Technology, Danvers, MA, USA) following the rapid protocol. After treatment, cells were lysed, lysates were applied to antibody-coated wells together with the HRP-conjugated detection antibody, and the signal was developed using TMB substrate and read at 450 nm. Details are provided in Supplementary Materials.

#### 4.2.11. In Vitro Human Liver Microsomal Stability

Compound **6** was incubated with pooled human liver microsomes (0.5 mg microsomal protein/mL) at 37 °C in phosphate buffer (pH 7.4). The reaction was initiated by adding NADPH, and aliquots were collected at predefined time points (0–60 min) and immediately quenched with ice-cold acetonitrile containing diazepam as an internal standard. After protein precipitation and centrifugation, the supernatants were analyzed by LC–MS/MS and the depletion of parent compound over time was used to determine the microsomal half-life and intrinsic clearance (in vitro). A complete step-by-step protocol and calculation workflow are provided in Supplementary Materials.

#### 4.2.12. Molecular Docking

Compound **6** was docked into tubulin (PDB: 4O2B), carbonic anhydrase IX (PDB: 3IAI), and carbonic anhydrase XII (PDB: 1JD0) using AutoDock Vina 1.2.0. Protein and ligand preparations were performed in AutoDock Tools, including hydrogen addition, charge assignment, and file conversion to PDBQT format. Binding poses were visualized and analyzed using Discovery Studio Visualizer. Further details are provided in Supplementary Materials.

#### 4.2.13. ADMET Prediction

The pharmacokinetic and safety-related properties of compound **6** were predicted in silico using ADMETLab 3.0. Key parameters related to physicochemical properties, absorption, distribution, metabolism, excretion, and toxicity were evaluated, and a bioavailability radar was generated to assess overall drug-likeness. Detailed prediction outputs and descriptors are provided in Supplementary Materials.

## Figures and Tables

**Table 1:** Selectivity index (SI) values of compound **6** compared with reference compounds CA-4 and SLC-0111 across different cancer cell lines, calculated as the ratio of IC_50_ in non-tumorigenic MCF-10A cells to IC_50_ in the corresponding cancer cells.

Compound	Selectivity Index (SI)			
	MDA-MB-231	HepG2	A549	HT-29
Compound **6**	114.9	1.93	10.53	12.08
CA-4	10.51	4.34	20.54	8.12
SLC-0111	2.36	1.57	1.30	2.82

**Table 2:** Inhibitory activity of compound **6** and CA-4 against tubulin polymerization, expressed as IC_50_ values (µM, mean ± SEM).

Compound	Tubulin Polymerization Inhibition IC_50_ ± SEM (µM)
Compound **6**	3.40 ± 0.09
CA-4	3.28 ± 0.14

**Table 3:** Inhibitory activity of compound **6** against human carbonic anhydrase isoforms I, II, IX, and XII, expressed as IC_50_ values (µM, mean ± SEM), in comparison with acetazolamide (AAZ) and SLC-0111.

Compound	CA Inhibition IC_50_ (µM) ± SEM			
	CA I	CA II	CA IX	CA XII
Compound **6**	5.483 ± 0.12	3.142 ± 0.08	0.102 ± 0.005	0.213 ± 0.004
AAZ	0.367 ± 0.02	0.153 ± 0.01	0.105 ± 0.01	0.029 ± 0.001
SLC-0111	1.36 ± 0.07	0.498 ± 0.04	0.048 ± 0.006	0.096 ± 0.008

**Table 4:** Selectivity index (SI) values of compound **6** relative to cytosolic carbonic anhydrase isoforms (CA I and CA II) versus tumor-associated isoforms (CA IX and CA XII), calculated from IC_50_ ratios.

Compound	Selectivity Index (SI)			
	CA I/CA IX	CA II/CA IX	CA I/CA XII	CA II/CA XII
Compound **6**	53.8	30.8	25.7	14.8
AAZ	3.5	1.5	12.7	5.3
SLC-0111	28.3	10.4	14.2	5.2

**Table 5:** Results of Western blot analysis of compound **6** using the MDA-MB-231 cancer cell line.

Compound	CA IX	CA XII	β-Actin
Compound **6**	2.090	2.820	√
DMSO (Control)	5.623	6.959	√

**Table 6:** Apoptosis and necrosis induction of compound **6** in the MDA-MB-231 cancer cell line.

Compound No.	Apoptosis			Necrosis
	Total	Early	Late	
Compound **6**	29.47	1.95	22.39	5.13
DMSO (Control)	3.31	0.88	0.14	2.29

**Table 7:** Effect of compound **6** on key intrinsic apoptotic markers (Bax, Bcl-2, and cytochrome c) in MDA-MB-231 cells. Protein levels are expressed as mean ± SEM, and fold changes are calculated relative to DMSO-treated control cells.

Compound	Bax (pg/µL) ± SEM	Fold Change	Bcl-2 (pg/µL) ± SEM	Fold Change	Cytochrome C (ng/µL) ± SEM	Fold Change
Compound **6**	505.28 ± 5.46	4.95	5.72 ± 0.08	0.320	0.680 ± 0.030	9.07
DMSO (Control)	102.09 ± 2.27	1	17.88 ± 0.11	1	0.075 ± 0.005	1

**Table 8:** Effect of compound **6** on caspase-9 and caspase-3 levels in MDA-MB-231 cells. Data are expressed as mean ± SEM, and fold changes are calculated relative to DMSO-treated control cells.

Compound	Caspase-3 (pg/µL) ± SEM	Fold Change	Caspase-9 (ng/µL) ± SEM	Fold Change
Compound **6**	456.5 ± 7.5	10.49	58.57 ± 1.44	10.4
DMSO (Control)	43.50 ± 1.5	1	5.625 ± 0.275	1

**Table 9:** % closure in HUVEC scratch assay after 72 h for vehicle control and compound **6**–treated cells.

Sample	% Closure
Compound **6**	65.185
DMSO (Control)	94.815

**Table 10:** Concentration-dependent inhibition (%) of VEGFR-2 (Tyr1175) phosphorylation in VEGF-stimulated HUVECs treated with compound **6**.

Compound	Concentration (µM)	VEGFR-2 (Tyr1175) Inhibition (%)
Compound **6**	0.01	6.73 ± 1.28
	0.1	23.29 ± 2.47
	1	38.84 ± 1.93

**Table 11:** In vitro human liver microsomal stability of compound **6**. Data are mean ± SD from two independent incubations (*n* = 2) per concentration. CLint, in vitro, was calculated using HLM protein = 0.5 mg/mL.

Start Conc. (µM)	t1/2 (h)	CLint (µL/min/mg)	% Remaining at 60 min
0.8	0.571 ± 0.028	40.53 ± 1.96	30.0 ± 1.7
4	0.883 ± 0.067	26.22 ± 2.00	44.9 ± 4.4
20	1.421 ± 0.079	16.28 ± 0.91	63.3 ± 0.7

## Data Availability

The original contributions presented in this study are included in the article; further inquiries can be directed to the corresponding author.

## Supplementary Materials

The following supporting information can be downloaded at https://www.mdpi.com/article/10.3390/molecules31060917/s1. Figure S1: ^1^H-NMR spectrum of the target compound (400 MHz, DMSO-*d*_6_); Figure S2: ^13^C-NMR spectrum of the target compound (100 MHz, DMSO-*d*_6_); Figure S3: Docking of the hydrolyzed E-isomer of compound **6** (thiol form) into hCA IX: (**A**) 2D interaction diagram and (**B**) 3D binding pose at the entrance of the catalytic site; Figure S4: Docking of the hydrolyzed E-isomer of compound **6** (thiol form) into hCA XII: (**A**) 2D interaction diagram and (**B**) 3D binding pose at the entrance of the catalytic site; Figure S5: Docking of the hydrolyzed E-isomer of compound **6** (thiol form) into tubulin: (**A**) 2D interaction diagram and (**B**) 3D binding pose at the colchicine binding site.
