# Supplementary material for: Discovery of a Novel Coumarin/Thiazole Chalcone Hybrid as a Potent Dual Inhibitor of Tubulin and Carbonic Anhydrases IX & XII with Promising Anti-Proliferative Activity
Source: Molecules. 2026 Mar 10;31(6):917. doi: 10.3390/molecules31060917 (PMC13028722; doi:10.3390/molecules31060917)
Supplement: Supplementary file 1 [file molecules-31-00917-s001.zip › molecules-4107795-supplementary.pdf]

Supporting information

Discovery of a Novel Coumarin/Thiazole Chalcone Hybrid as a Potent Dual Inhibitor of Tubulin and Carbonic Anhydrases IX & XII with Promising Anti-Proliferative Activity

Basima A. A. Saleem [1], Ashraf A. Qurtam [2], Mohamed Ahmed [3], Raed Fanoukh Aboqader Al-Aouadi [4], Ali Abdulrazzaq Abdulhussein Alrikabi [5], Helal F. Hetta [6], Stefan Bräse [7,*], Ghallab Alotaibi [8], Abdullah Alkhammash [8] and Sara Mahmoud Farhan [9,*]

[1] Department of Chemistry, College of Science, University of Mosul, Mosul 41001, Iraq
[2] Biology Department, College of Science, Imam Mohammad Ibn Saud Islamic University (IMSIU), Riyadh 11623, Saudi Arabia
[3] College of Medicine, Dhofar University, Salalah 122, Oman
[4] College of Medicine, Al-Ayen Iraqi University, AUIQ, An Nasiriyah 64001, Iraq
[5] College of Medicine, University of Thi-Qar, Nasiriyah 64001, Iraq
[6] Division of Microbiology, Immunology and Biotechnology, Department of Natural Products and Alternative Medicine, Faculty of Pharmacy, University of Tabuk, Tabuk 71491, Saudi Arabia
[7] Institute of Biological and Chemical Systems—Functional Molecular Systems (IBCS-FMS), Karlsruhe Institute of Technology (KIT), Kaiserstrasse 12, 76131 Karlsruhe, Germany
[8] Department of Pharmacology, College of Pharmacy, Al-Dawadmi Campus, Shaqra University, Shaqra 11961, Saudi Arabia
[9] Department of Microbiology and Immunology, Faculty of Pharmacy, Deraya University, New Minia 61768, Egypt
* Correspondence: stefan.braese@kit.edu (S.B.); sara.mahmoud@deraya.edu.eg (S.M.F.)

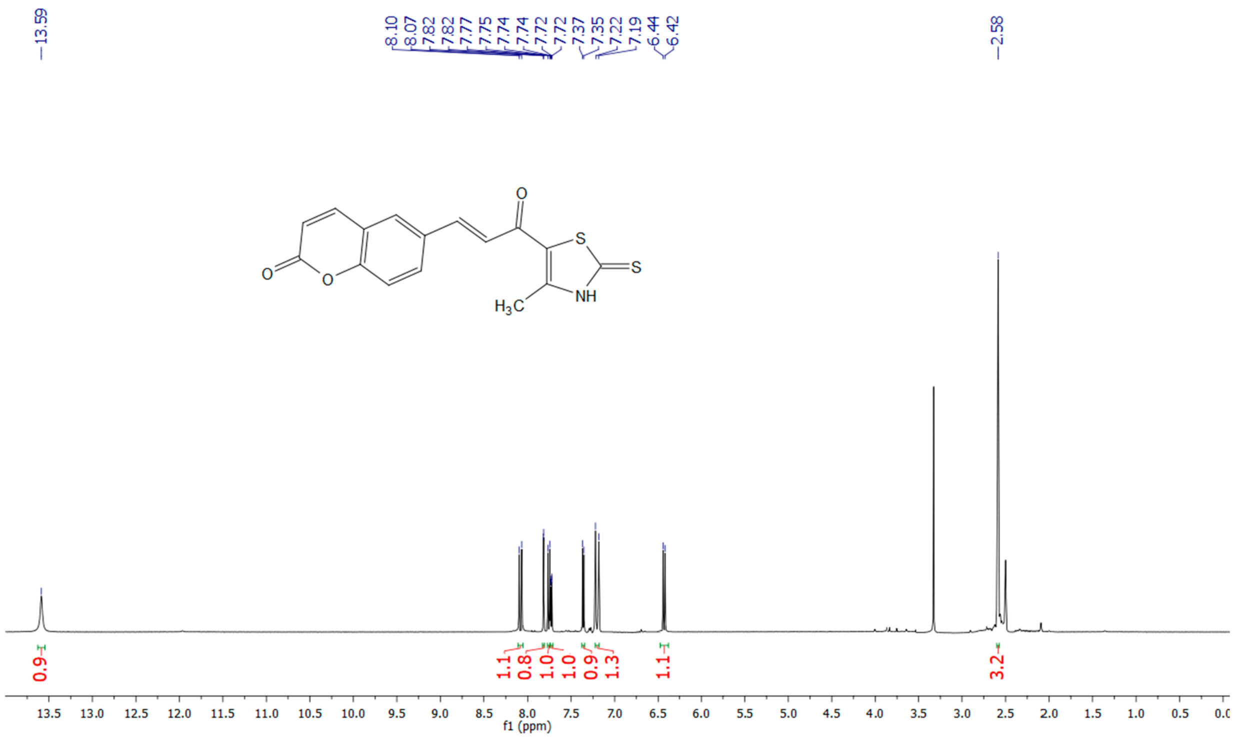

Figure S1. ^{1}H-NMR spectrum of the target compound (400 MHz, DMSO-d_6)

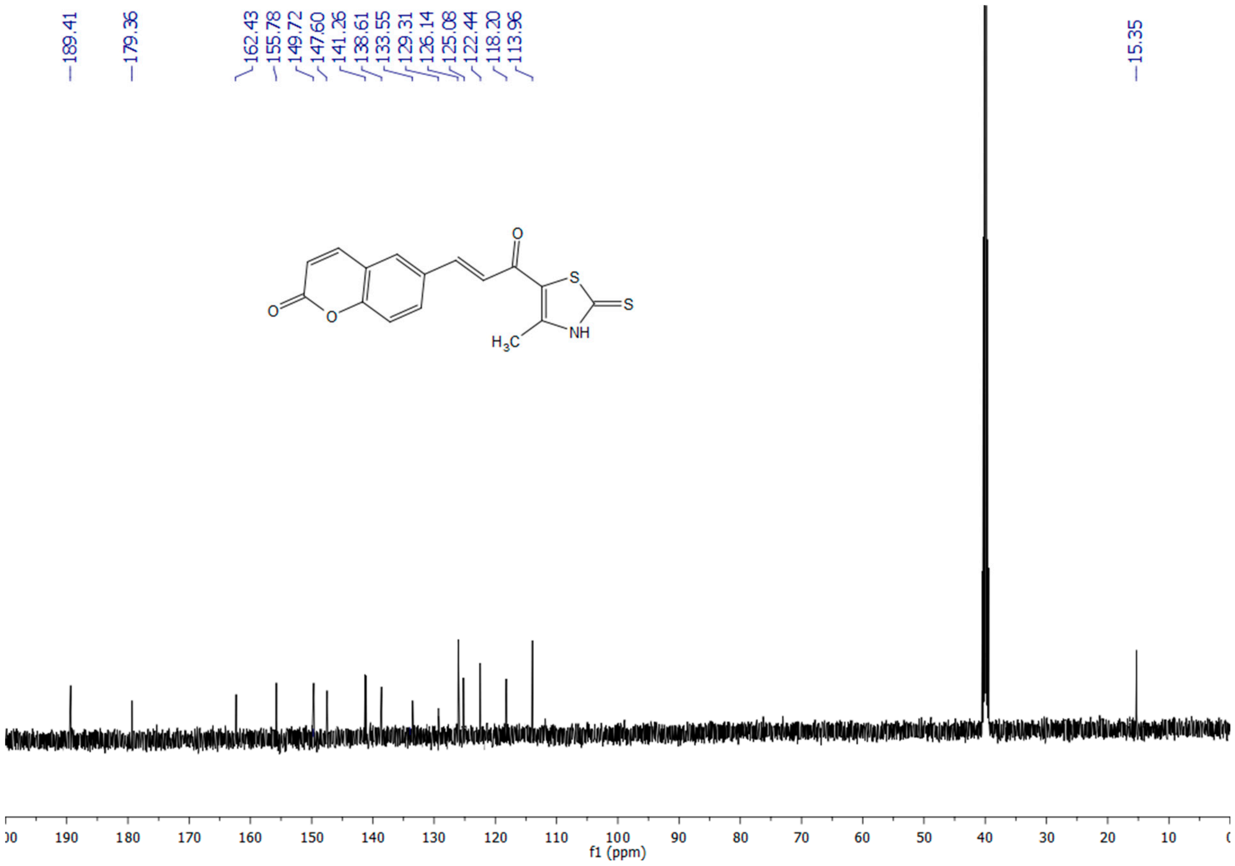

Figure S2. ^{13}C-NMR spectrum of the target compound (100 MHz, DMSO-*d*6)

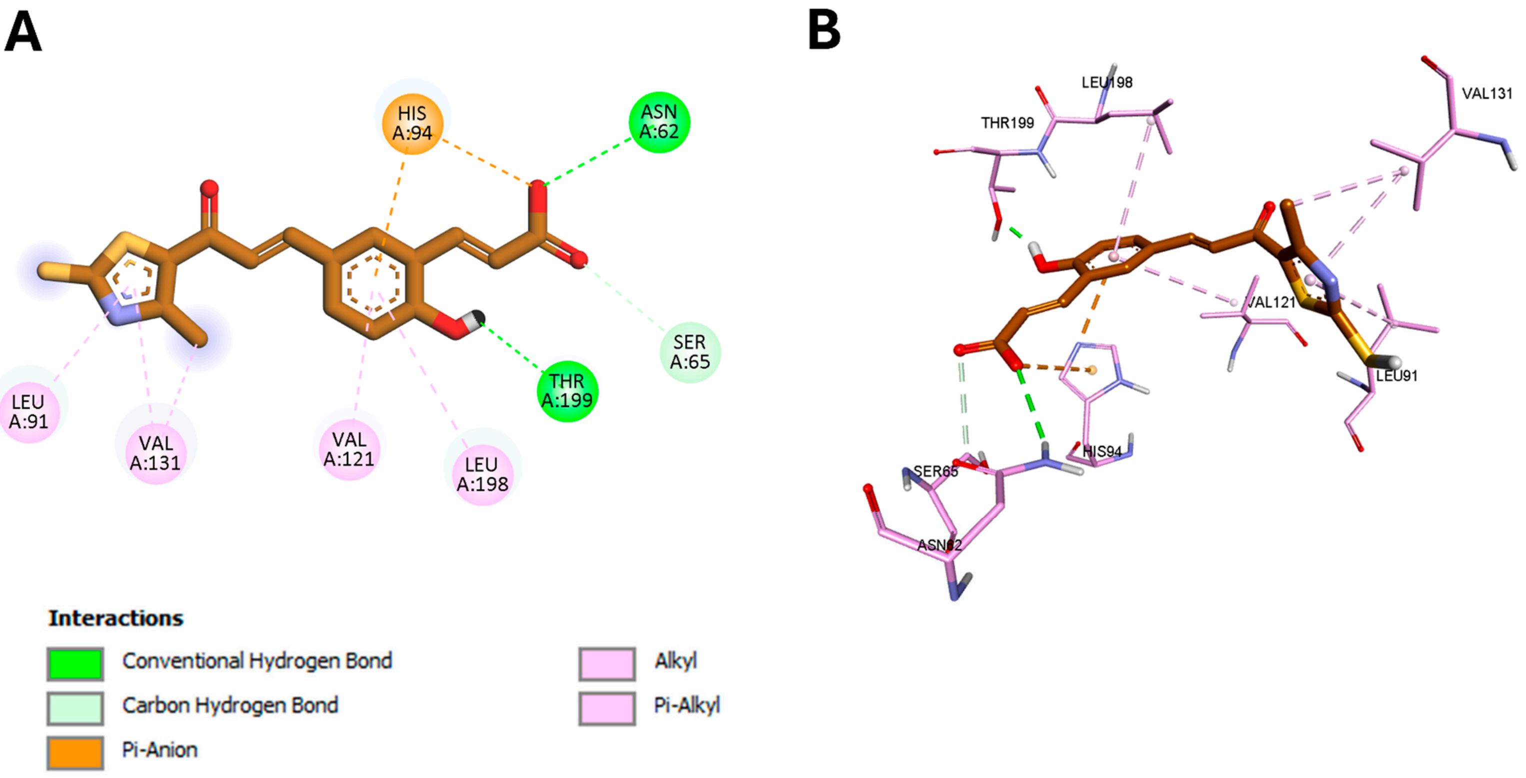

Figure S3. Docking of the hydrolyzed E-isomer of compound **6** (thiol form) into hCA IX: **(A)** 2D interaction diagram and **(B)** 3D binding pose at the entrance of the catalytic site.

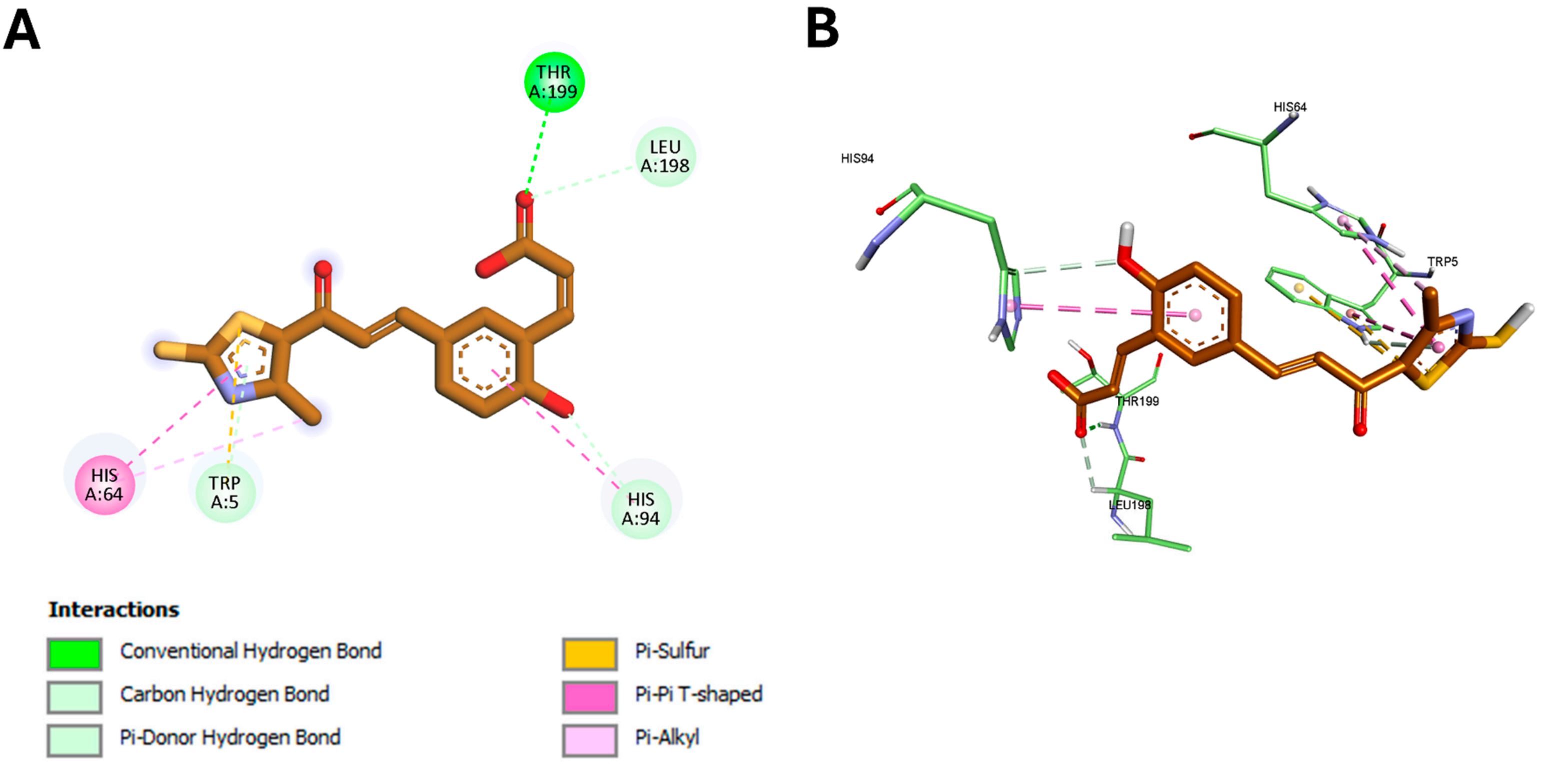

Figure S4. Docking of the hydrolyzed E-isomer of compound **6** (thiol form) into hCA XII: **(A)** 2D interaction diagram and **(B)** 3D binding pose at the entrance of the catalytic site.

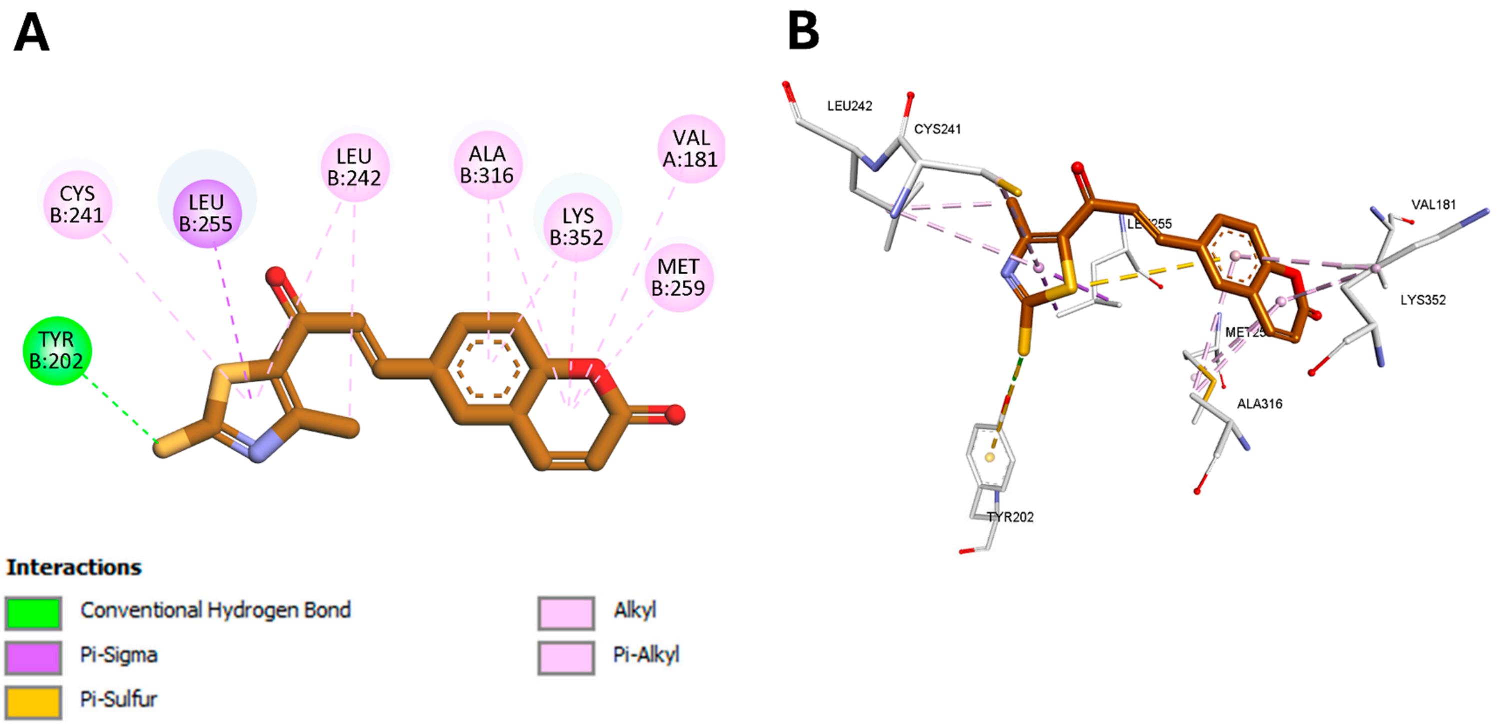

Figure S5. Docking of the hydrolyzed E-isomer of compound **6** (thiol form) into tubulin: **(A)** 2D interaction diagram and **(B)** 3D binding pose at the colchicine binding site.

4. Experimental

4.1. Chemistry

General Information

All reagents and solvents were of general purpose or analytical grade and purchased from Sigma Aldrich Ltd, Fisher Scientific, Fluka and Acros. ^1H- and ^{13}C-NMR spectra were recorded with a Bruker Avance III spectrometer operating at 400, 100 MHz respectively, with Me_4Si as internal standard and DMSO-d_6 as a solvent. Elemental analysis was performed by the regional center for mycology and biotechnology (Cairo, Egypt). TLC was carried out on precoated silica plates (Keisel gel 60 F254, BDH) using Hexane: Ethyl acetate, 1 : 2, v/v. Compounds were visualized by illumination under UV light (254 nm). Melting points were determined on an electrothermal instrument and are uncorrected. All solvents were dried prior to use and stored over 4 Å molecular sieves, under nitrogen. All the compounds were ≥ 95% pure.

4.2. Biological evaluation

2.1. Antiproliferative Assay

The cytotoxic effect of compound 6 was evaluated in comparison with the reference tubulin inhibitor combretastatin A-4 (CA-4) and the selective carbonic anhydrase IX inhibitor SLC-0111 against four human cancer cell lines: MDA-MB-231 (triple-negative breast carcinoma), HepG2 (hepatocellular carcinoma), A549 (lung carcinoma), and HT-29 (colon adenocarcinoma), as well as the non-tumorigenic human mammary epithelial cell line MCF-10A. Cells were cultured in their respective recommended media supplemented with 10% fetal bovine serum and 1% penicillin–streptomycin and maintained at 37 °C in a humidified atmosphere containing 5% CO_2. Cell viability was assessed using the MTT assay, following the protocol provided with the In Vitro Toxicology Assay Kit, MTT Based (Sigma-Aldrich, TOX-1). Briefly, cells were seeded in 96-well plates at a density of 5×10^3 cells/well and allowed to adhere overnight. The following

day, cells were treated with increasing concentrations of compound 6, CA-4, or SLC-0111 and incubated for 48 h. Subsequently, 10 µL of reconstituted MTT solution (5 mg/mL in serum-free, phenol-red-free medium) was added to each well and incubated for 3 h at 37 °C. The resulting formazan crystals were dissolved by adding 100 µL of the MTT solubilization solution, and the plates were gently shaken to ensure complete solubilization. Absorbance was measured at 570 nm with a reference wavelength of 690 nm using a microplate reader. Cell viability was expressed as a percentage relative to untreated control cells. All experiments were performed in triplicate, and IC_{50} values were determined using linear regression analysis.

2.2. Tubulin polymerization assay

The effect of compound 6 on tubulin polymerization was evaluated in comparison with the reference microtubule destabilizing agent combretastatin A-4 (CA-4), using the Tubulin Polymerization Assay Kit (Fluorescence-based, Cytoskeleton, Inc., Cat. No. BK011P), according to the manufacturer's protocol. This fluorescence-based assay monitors the polymerization of purified porcine brain tubulin by detecting the enhanced fluorescence signal resulting from the incorporation of a fluorescent reporter into assembling microtubules. Compound 6 and CA-4 were each prepared as 10× stock solutions in DMSO and diluted to the desired concentrations in assay buffer. Each well of a pre-warmed 96-well black flat-bottom plate received 5 µL of compound solution or vehicle control. The polymerization reaction was initiated by adding 50 µL of the tubulin reaction mix (2 mg/mL tubulin in 80 mM PIPES, pH 6.9, 2.0 mM $MgCl_2$, 0.5 mM EGTA, 1.0 mM GTP, and 15% glycerol). The plate was immediately transferred to a temperature-controlled microplate reader pre-equilibrated at 37 °C. Tubulin polymerization was monitored kinetically by recording fluorescence at 360 nm excitation and 420 nm emission every minute for 60 minutes. Control wells included vehicle (DMSO), paclitaxel (as a polymerization enhancer), and vinblastine (as a polymerization inhibitor). The inhibitory effects of compound 6 and CA-4 were determined by analyzing

changes in polymerization kinetics, including the nucleation lag phase and the maximal polymerization rate (Vmax). IC_{50} values were calculated from the fluorescence curves using linear regression analysis.

2.3. Evaluation of Carbonic anhydrase I, II, IX, and XII inhibition

The inhibitory activity of compound 6 against human carbonic anhydrase (CA) isoforms I, II, IX, and XII was assessed in comparison with the reference CA inhibitors acetazolamide (AAZ) and SLC-0111 using the Carbonic Anhydrase Inhibitor Screening Kit (Colorimetric, BioVision, Cat. No. K473-100), following the manufacturer's protocol. This assay is based on the esterase activity of active CA enzymes on a chromogenic substrate, which generates a colorimetric signal detectable at 405 nm. Recombinant human CA isoforms I, II, IX, and XII were incubated with varying concentrations of compound 6, AAZ, or SLC-0111 in 96-well plates. Each well contained 80 µL of CA assay buffer, 5 µL of reconstituted CA enzyme, and 10 µL of inhibitor solution (dissolved in DMSO), followed by a 10-minute incubation at room temperature. The same pre-incubation time (10 min) was applied for all CA isoforms and for compound 6, AAZ, and SLC-0111. The reaction was initiated by the addition of 5 µL of CA substrate, and absorbance was recorded kinetically at 405 nm for 60 minutes using a microplate reader. The enzymatic activity in the presence of compound 6, AAZ, or SLC-0111 was compared to enzyme control wells lacking inhibitor. The relative activity and percentage inhibition were calculated for each compound. IC_{50} values were determined from the resulting dose–response curves using linear regression analysis.

2.4. Western Blotting

Protein expression levels of carbonic anhydrase IX (CAIX) and carbonic anhydrase XII (CAXII) were evaluated by western blotting. Following compound treatment, MDA-MB-231 cells were washed with ice-cold PBS and lysed using RIPA buffer supplemented with protease inhibitors and PMSF. Lysates were incubated on ice, sonicated briefly, and clarified by centrifugation

(14,000 × g, 20 min, 4 °C). Protein concentration was determined using a BCA assay, and samples were normalized to equal protein content prior to electrophoresis.

Proteins (20–30 µg per lane) were resolved by SDS-PAGE on gradient polyacrylamide gels and transferred onto PVDF membranes using wet transfer conditions. Membranes were blocked in 5% non-fat milk in TBST and incubated with primary antibodies against CAIX (~54 kDa) and CAXII (~45 kDa) overnight at 4 °C. β-Actin (~42 kDa) was used as a loading control.

After washing, membranes were incubated with HRP-conjugated secondary antibodies and developed using enhanced chemiluminescence substrate. Signals were visualized using a digital imaging system, and band intensities were quantified by densitometry using ImageJ or equivalent software. Target protein expression was normalized to β-actin and expressed relative to untreated control samples.

2.5. Cell cycle analysis

MDA-MB-231 cells were seeded and treated with compound 6 at the indicated concentration and exposure time. After treatment, both adherent and floating cells were collected to avoid loss of mitotic and apoptotic populations, washed with PBS, and pelleted by centrifugation (500 × g, 5 min). Cells were fixed in 66% ice-cold ethanol added dropwise while vortexing and stored at 4 °C for at least 2 h to ensure permeabilization and stabilization of DNA content. Prior to analysis, cells were washed with PBS and resuspended in propidium iodide/RNase staining solution followed by incubation for 20–30 min at 37 °C in the dark. Samples were analyzed using a flow cytometer equipped with a 488 nm laser, and PI fluorescence was collected in the FL2 channel. Debris and aggregates were excluded by forward and side scatter gating. DNA histograms were generated, and the percentages of cells in G0/G1 (2N), S (2N–4N), and G2/M (4N) phases were quantified using flow cytometry analysis software. Results were expressed as mean ± SD from independent experiments.

2.6. Apoptosis assay

Apoptosis induced by compound 6 in MDA-MB-231 cells was evaluated using Annexin V-FITC/propidium iodide (PI) dual staining followed by flow cytometric analysis. Briefly, MDA-MB-231 cells were seeded and treated with compound 6 under the indicated experimental conditions. After treatment, both floating and adherent cells were collected to avoid underestimation of apoptotic populations, washed twice with cold phosphate-buffered saline, and centrifuged to obtain a single-cell suspension. Cells (1–5 × 10^5) were resuspended in 500 µL of binding buffer and incubated with Annexin V-FITC (5 µL) and PI (5 µL) for 5 min at room temperature in the dark to allow detection of phosphatidylserine externalization and membrane integrity loss. Samples were subsequently analyzed by flow cytometry using 488 nm excitation, with FITC fluorescence detected in the FL1 channel and PI fluorescence detected in the FL2 channel. Appropriate forward- and side-scatter gating was applied to exclude debris and cell aggregates, and quadrant analysis was performed to discriminate viable (Annexin V^-/PI^-), early apoptotic (Annexin V^+/PI^-), late apoptotic (Annexin V^+/PI^+), and necrotic (Annexin V^-/PI^+) cell populations. The percentages of cells in each quadrant were quantified using flow cytometry analysis software, and results were expressed as mean ± SD from independent experiments.

2.7. Effect on *BAX* expression levels

The level of BAX protein expression following treatment with compound 6 was determined using the Human BAX ELISA Kit (DRG International, Inc., Cat. No. EIA-4487), according to the manufacturer's instructions. This sandwich-based immunoassay utilizes a monoclonal antibody specific for human BAX-α to capture and quantify the protein in cell lysates. Cells were lysed using the provided Cell Lysis Buffer, freshly supplemented with phenylmethylsulfonyl fluoride (PMSF, 1 mM) and protease inhibitor cocktail (PIC, 0.5 µL/mL). Lysates were incubated on ice, vortexed briefly, and centrifuged at 16,000 rpm for 15 minutes at 4 °C. The resulting supernatants were collected and diluted using the kit's Assay Buffer. A standard curve was generated using BAX-α standards at concentrations of 62.5, 125, 250, 500, 1000, and 2000 ng/mL. The

corresponding net absorbance values, after subtraction of the average blank OD (0.023), were 0.090, 0.243, 0.390, 0.765, 1.159, and 2.378, respectively. Absorbance was measured at 450 nm using a microplate reader, with a reference wavelength of 570–590 nm. BAX concentrations in the treated samples were calculated based on the linear regression equation derived from the standard curve. This assay provided a sensitive and quantitative measure of BAX protein modulation in response to compound 6.

2.8. Effect on *Bcl-2* expression levels

The Bcl-2 protein levels following treatment with compound 6 were quantified using the Zymed® Bcl-2 ELISA Kit (Cat. No. 99-0042), following the manufacturer's instructions. This assay employs a sandwich ELISA format in which human Bcl-2 is captured by a monoclonal antibody immobilized on a microwell plate and subsequently detected using a biotin-conjugated anti-Bcl-2 antibody, followed by Streptavidin-HRP and TMB substrate for colorimetric detection. Cells were lysed in the provided Lysis Buffer at a density of 5×10^6 cells/mL and incubated at room temperature for 1 hour with gentle shaking. Lysates were clarified by centrifugation at 1000 × g for 15 minutes, and the supernatants were collected and used immediately or stored at –80 °C until analysis. For the assay, 20 µL of each sample was mixed with 80 µL of Sample Diluent in designated wells, followed by the addition of 50 µL of diluted biotin-conjugated antibody. After a 2-hour incubation at room temperature, plates were washed and incubated for 1 hour with diluted Streptavidin-HRP. TMB substrate was then added, and color development was allowed for 15 minutes before the reaction was stopped with phosphoric acid. Absorbance was measured at 450 nm using a microplate reader, with optional correction between 610–650 nm. A standard curve was generated using Bcl-2 standards at concentrations of 1, 2, 4, 8, 16, and 32 ng/mL. The corresponding absorbance values ranged from approximately 0.102 to 2.463, with a blank reading of 0.022. Net absorbance values were obtained by subtracting the blank, and Bcl-2 concentrations in test samples were calculated by linear regression analysis based on the standard curve.

2.9. Effect of *caspase-3* activity

The level of active caspase-3 protein following treatment with compound 6 was quantified using the Human Active Caspase-3 ELISA Kit (Invitrogen, Cat. No. KHO1091), according to the manufacturer's instructions. This assay is based on a solid-phase sandwich ELISA principle, in which an anti-human caspase-3 monoclonal antibody is pre-coated onto a microplate. Cell lysates or standards are captured by this immobilized antibody, followed by the addition of a rabbit polyclonal detection antibody specific for active caspase-3 cleaved at Asp175/Ser176. Detection is subsequently achieved using HRP-conjugated anti-rabbit IgG and TMB substrate. Cell lysates were prepared using protease inhibitor-supplemented cell extraction buffer and clarified by centrifugation. Samples were diluted in Standard Diluent Buffer and added to the wells together with a standard curve of human active caspase-3. The standard concentrations were 39, 78, 156, 313, 625, 1250, and 2500 ng/mL, yielding optical density (OD) readings in the range of approximately 0.203 to 2.708. A blank absorbance value of 0.018 was subtracted from each standard OD to obtain the corresponding net absorbance values, which were used to construct the standard calibration curve. Following incubation and sequential wash steps, the detection antibody, HRP conjugate, and TMB substrate were added. After color development, the reaction was terminated with stop solution, and absorbance was measured at 450 nm using a microplate reader. The concentration of active caspase-3 in treated samples was determined from the standard curve using linear regression analysis, and values were corrected for the applied dilution factor.

2.10. Effect of *caspase-9* activity

The concentration of human caspase-9 protein following treatment with compound 6 was quantified using the Human Caspase-9 ELISA Kit (Thermo Fisher Scientific, Cat. No. BMS2025), following the manufacturer's protocol. This solid-phase sandwich ELISA utilizes a monoclonal antibody pre-coated onto a 96-well microplate to capture caspase-9 from biological samples. A rabbit polyclonal detection antibody specific to human caspase-9 is subsequently

added, followed by HRP-conjugated anti-rabbit IgG and TMB substrate for colorimetric detection. The intensity of the yellow reaction product formed after stopping the reaction with phosphoric acid is proportional to the amount of caspase-9 present and is measured at 450 nm.

Cell lysates were prepared in 1× lysis buffer supplied with the kit, using approximately 5×10^6 cells/mL, and incubated for 60 minutes at room temperature with gentle agitation. Lysates were then centrifuged at 1,000 × g for 15 minutes, and the clarified supernatants were collected and either analyzed immediately or stored at –80 °C until use. Samples were diluted 1:2 with Sample Diluent and added to the microplate together with a standard curve prepared by 1:2 serial dilution of the reconstituted standard. Standard concentrations of 1.6, 3.1, 6.3, 12.5, 25, 50, and 100 ng/mL yielded optical density (OD) readings of 0.146, 0.181, 0.223, 0.265, 0.521, 0.753, and 1.612, respectively, with a blank absorbance of 0.040. Net OD values were calculated by subtracting the blank from each standard reading, and these values were used to construct the standard calibration curve. The concentration of caspase-9 in the treated samples was determined using linear regression analysis based on the standard curve, and final concentrations were corrected for the sample dilution factor.

2.11. Effect on *cytochrome c* expression levels

The intracellular release of cytochrome c was quantified using the Human Cytochrome c ELISA Kit (Thermo Fisher Scientific, Catalog No. BMS263), according to the manufacturer's protocol. In brief, cells were harvested following treatment, washed with ice-cold PBS, and lysed using the provided lysis buffer (diluted 1:10) at a concentration of 1.5×10^6 cells/mL. The lysates were incubated at room temperature for one hour with gentle agitation, followed by centrifugation at 200 × g for 15 minutes. The resulting supernatants were collected, diluted 1:50 in assay buffer, and stored at –70 °C until analysis. A standard curve was generated using seven serial two-fold dilutions of reconstituted cytochrome c standard, spanning concentrations from 5.00 to 0.08 ng/mL, with assay buffer serving as the blank. The optical density (OD) values at 450 nm for

these standards were 2.141, 1.233, 0.632, 0.258, 0.093, 0.076, and 0.050 respectively, with the blank reading at 0.029. Standards and samples were added in duplicate to microwells pre-coated with monoclonal anti-human cytochrome c antibodies, followed by incubation with a biotin-conjugated detection antibody and subsequent binding of streptavidin–HRP. After the addition of TMB substrate and a 10-minute incubation, the reaction was stopped with 1 M phosphoric acid. Absorbance was recorded at 450 nm using a microplate reader. Cytochrome c concentrations in experimental samples were determined by linear regression analysis based on the standard curve, and values were corrected for the initial dilution factor. All measurements were performed in duplicate.

2.12. Wound healing (scratch) assay on HUVEC

HUVEC cells were maintained under standard culture conditions (37 °C, 5% CO_2) and seeded into multiwell plates to reach ~90–100% confluence within 24–48 h. To generate the wound, the plate underside was marked to allow imaging of the same field over time, then a straight scratch was made across the monolayer using a sterile 200-µL pipette tip held perpendicular to the plate; where feasible, ≥2 scratches were created per well. Detached cells were removed by washing 1–2× with PBS, followed by addition of fresh medium containing the test compound (vehicle control in matched solvent). If migration-focused conditions were required, proliferation was minimized using serum reduction and/or mitomycin-C pretreatment as indicated. Phase-contrast images were acquired at t = 0 immediately after treatment addition and at the indicated intervals (e.g., 6–48 h, depending on migration rate), using 4×–10× objectives while maintaining sterility and minimizing time outside the incubator. Image analysis was performed in ImageJ/Fiji using the MRI Wound Healing Tool (or manual width measurements at multiple positions per wound), keeping identical scale settings across conditions. Wound closure was calculated as: % closure = $[(Width_0 - Width_t) / Width_0] \times 100$, and values were reported as mean ± SD from replicate wells

(≥3 technical replicates per condition recommended) with appropriate statistical comparisons versus control.

2.13. Inhibition of VEGFR-2 phosphorylation in HUVEC cells

Phosphorylated VEGFR-2 (Tyr1175) levels were quantified using the PathScan® RP Phospho-VEGFR-2 (Tyr1175) Sandwich ELISA Kit (#7335), which is based on a solid-phase sandwich immunoassay that captures VEGFR-2 and detects phosphorylation at Tyr1175 through a rapid single incubation of cell lysate with an HRP-conjugated detection antibody on antibody-coated microwells. Microwell strips were equilibrated to room temperature prior to use, and unused strips were resealed with desiccant and stored at 4 °C. The detection antibody was reconstituted in HRP diluent, gently mixed for approximately 5 min, and diluted to the working concentration immediately before use. Wash buffer and cell lysis buffer were prepared by diluting the respective concentrated stocks to 1×, and protease/phosphatase inhibitors together with PMSF were freshly added to the lysis buffer. TMB substrate and stop solution were allowed to reach room temperature before use. For sample preparation, adherent cells at ~80–90% confluence were treated under the indicated conditions, washed with ice-cold PBS, and lysed in ice-cold lysis buffer supplemented with inhibitors. Cells were incubated on ice for ~5 min, scraped into tubes, briefly sonicated, and centrifuged (14,000 rpm, 10 min, 4 °C) to obtain clarified lysates, which were stored at −80 °C until analysis. During the ELISA procedure, lysates were used either undiluted or diluted in lysis buffer to achieve the desired protein concentration. For each well, 50 µL of lysate was added followed by 50 µL of detection antibody, and plates were incubated for 1 h at room temperature on a plate shaker (~400 rpm). Wells were washed four times with wash buffer, after which 100 µL of TMB substrate was added and incubated in the dark for approximately 15 min before addition of 100 µL stop solution. Absorbance was measured at 450 nm within 30 min of stopping the reaction.

2.14. In Vitro Human Liver Microsomal Stability

Metabolic stability of compound 6 was evaluated using pooled human liver microsomes (HLM) under NADPH-supported oxidative conditions. Briefly, incubations were prepared in 100 mM potassium phosphate buffer (pH 7.4) containing HLM at a final microsomal protein concentration of 0.5 mg/mL (optionally supplemented with $MgCl_2$ to 3 mM). Compound 6 was added from a DMSO stock to give final concentrations of 0.8, 4, and 20 µM while keeping the final DMSO content low (≤0.1%, v/v). The incubation mixtures were pre-equilibrated at 37 °C, and reactions were initiated by the addition of NADPH (final ~1 mM) or an NADPH-regenerating system; the time of cofactor addition was defined as t = 0. At predetermined time points over 0–60 min (e.g., 0, 5, 10, 15, 30, 45, and 60 min), aliquots were removed and immediately quenched with ice-cold acetonitrile containing diazepam as an internal standard to precipitate proteins and terminate enzymatic activity. Quenched samples were vortex-mixed, kept on ice, and centrifuged to pellet precipitated protein, after which the clarified supernatants were transferred for LC–MS/MS analysis. Parent compound depletion was quantified as the peak-area ratio of compound 6 to the diazepam internal standard at each time point. For each incubation, log10-transformed peak-area ratios were plotted against time expressed in hours and fitted by linear regression to obtain the slope (m); the apparent first-order depletion rate constant was calculated as k (h^{-1}) = −2.303 × m and the microsomal half-life was calculated as t1/2 (h) = 0.693/k. Intrinsic clearance in vitro was calculated using the microsomal protein concentration as CLint,in vitro (µL/min/mg) = 1000 × [k/60]/0.5, where the factor 60 converts h^{-1} to min^{-1} and 0.5 mg/mL is the microsomal protein concentration used in the assay. All incubations were performed in duplicate for each concentration (n = 2), and results were reported as mean ± SD for t1/2 and CLint,in vitro.

3. Molecular Modeling

3.1. Molecular docking

The crystal structures of tubulin-colchicine complex (PDB code: 4O2B), human carbonic anhydrase IX (PDB code: 3IAI) and human carbonic anhydrase XII (PDB code: 1JD0) were downloaded from the Protein Data Bank. Structure of compound 6 was drawn and optimized using MarvinSketch and Avogadro molecular editors. The proteins were prepared using autodock tools where the co-crystallized water molecules and colchicine were removed then kollman charges and polar hydrogens were added. The grid coordinates for tubulin were set to 15.951x66.804x43.33 for x, y and z axes, respectively with grid dimensions of 80x80x80. Autodock vina was used for molecular docking and the best docking poses were visualized using Discovery Studio Visualizer.

3.2. ADMET predictions

The pharmacokinetic properties of compound 6 were predicted using the SwissADME web tool (http://www.swissadme.ch). The SMILES notation of the compound was input into the platform to evaluate key absorption, distribution, metabolism, and excretion (ADME) parameters. These included gastrointestinal (GI) absorption, blood-brain barrier (BBB) permeability, P-glycoprotein substrate prediction, cytochrome P450 enzyme inhibition, and physicochemical descriptors such as lipophilicity (LogP), solubility (LogS), and topological polar surface area (TPSA). Drug-likeness was also assessed based on *Lipinski's rule of five* and related filters.
